# Human Huntington’s disease pluripotent stem cell-derived microglia develop normally but are abnormally hyper-reactive and release elevated levels of reactive oxygen species

**DOI:** 10.1186/s12974-021-02147-6

**Published:** 2021-04-19

**Authors:** Grace C. O’Regan, Sahar H. Farag, Caroline S. Casey, Alison Wood-Kaczmar, Jennifer M. Pocock, Sarah J. Tabrizi, Ralph Andre

**Affiliations:** 1 Huntington’s Disease Centre, Department of Neurodegenerative Disease, UCL Queen Square Institute of Neurology, University College London, WC1N 3BG, London, UK; 2 Department of Neuroinflammation, UCL Queen Square Institute of Neurology, University College London, WC1N 1PJ, London, UK

**Keywords:** Neurodegeneration, Huntington’s disease, Pluripotent stem cells, Microglia, Striatal neurons, Cytokines, Reactive oxygen species

## Abstract

**Background:**

Neuroinflammation may contribute to the pathogenesis of Huntington’s disease, given evidence of activated microglia and elevated levels of inflammatory molecules in disease gene carriers, even those many years from symptom onset. We have shown previously that monocytes from Huntington’s disease patients are hyper-reactive to stimulation in a manner dependent on their autonomous expression of the disease-causing mutant HTT protein. To date, however, whether human microglia are similarly hyper-responsive in a cell-autonomous manner has not been determined.

**Methods:**

Microglial-like cells were derived from human pluripotent stem cells (PSCs) expressing mutant HTT containing varying polyglutamine lengths. These included lines that are otherwise isogenic, such that any observed differences can be attributed with certainty to the disease mutation itself. Analyses by quantitative PCR and immunofluorescence microscopy respectively of key genes and protein markers were undertaken to determine whether Huntington’s disease PSCs differentiated normally to a microglial fate. The resultant cultures and their supernatants were then assessed by various biochemical assays and multiplex ELISAs for viability and responses to stimulation, including the release of pro-inflammatory cytokines and reactive oxygen species. Conditioned media were applied to PSC-derived striatal neurons, and vice versa, to determine the effects that the secretomes of each cell type might have on the other.

**Results:**

Human PSCs generated microglia successfully irrespective of the expression of mutant HTT. These cells, however, were hyper-reactive to stimulation in the production of pro-inflammatory cytokines such as IL-6 and TNFα. They also released elevated levels of reactive oxygen species that have neurotoxic potential. Accompanying such phenotypes, human Huntington’s disease PSC-derived microglia showed increased levels of apoptosis and were more susceptible to exogenous stress. Such stress appeared to be induced by supernatants from human PSC-derived striatal neurons expressing mutant HTT with a long polyglutamine tract.

**Conclusions:**

These studies show, for the first time, that human Huntington’s disease PSC-derived microglia are hyper-reactive due to their autonomous expression of mutant HTT. This provides a cellular basis for the contribution that neuroinflammation might make to Huntington’s disease pathogenesis.

**Supplementary Information:**

The online version contains supplementary material available at 10.1186/s12974-021-02147-6.

## Background

Huntington’s disease (HD) is a fatal, autosomal dominant neurodegenerative disorder characterised by progressive cognitive, psychiatric and motor impairments, and widespread neuronal degeneration throughout the brain [1]. It is caused by a CAG triplet repeat expansion in exon 1 of the gene encoding huntingtin (*HTT*), resulting in a pathogenic expanded stretch of thirty six or more glutamine residues in the N-terminal region of the HTT protein [2]. HTT is expressed ubiquitously, however, and as such, mutant (m)HTT in non-neuronal cells may contribute to HD neuropathology and indeed have effects beyond the brain. For example, systemic immune cells express mutant HTT in a manner that correlates with the disease burden of HD patients [3] and various immune system abnormalities have been shown in Huntington’s disease gene carriers [4]. These include altered levels of circulating innate immune proteins that correlate with disease progression and can be detected years before disease onset [5–8]. Various evidence, moreover, suggests that the innate immune system may play a disease-modifying role in HD pathogenesis [9–14].

Myeloid cells comprising circulating monocytes and tissue-resident macrophages are the likely effector cells of innate immune system phenotypes in HD. Monocytes from HD patients are hyper-reactive in response to lipopolysaccharide (LPS), a phenotype replicated in mouse mutant HTT-expressing tissue-resident macrophage populations [6, 15, 16]. Such cells also exhibit functional deficits in their migratory and phagocytic capabilities [15, 17]*.* Microglia, the tissue-resident macrophages of the central nervous system (CNS), appear activated in the HD brain [18, 19]. This correlates with disease severity in symptomatic patients [20] and predicts disease onset in gene carriers for whom symptoms have not yet commenced [21, 22]. Activated microglia appear proximal to degenerating neurons and white matter, which are key pathological events in HD [23, 24]. In a transgenic mouse model of Huntington’ disease, depleting the numbers of microglia prevents the extracellular matrix changes and loss of striatal volume associated with disease progression [25].

HD-related differences in myeloid cell function occur by the cell-autonomous effects of mutant HTT expression, rather than merely as a secondary consequence of neuronal pathology [26]. For example, the hyper-reactive phenotype of human HD macrophages is reversed following siRNA-mediated HTT expression lowering [27, 28]. The mechanisms underlying these effects of mutant HTT may include a resting transcriptional dysfunction that is responsible for the phenotypic changes seen once the cells are activated, and/or interactions with signalling pathways known to drive inflammatory responses, such as that associated with the transcription factor, NFκB [27, 29]. Whether such phenotypes and mechanisms occur in human microglia, however, which comprise the macrophage population most proximal to the core sites of HD pathology in the CNS, has not yet been determined.

Human pluripotent stem cells (PSCs) and their differentiation to specific cell subtypes have enormous potential to model the tissues affected in human disease on a species-matched genetic background. Indeed, HD human PSC lines expressing HTT with various polyglutamine tract lengths have been derived by several means, with multiple mutant HTT-dependent effects, most commonly in cultures differentiated to a greater or lesser extent towards a neuronal fate, having been described [30–36]. No data regarding human HD PSC-derived microglia have been published to date.

Here, we sought to assess the effects of mutant HTT on the differentiation, viability and function of human PSC-derived microglia. Given that the genetic backgrounds of PSC donors are known to influence transcription and the differentiation of the resulting cell lines [37–39], and the use of isogenic or genetically matched PSC lines is being increasingly recommended as best practice for the robust use of such models [40], we have utilised here panels of isogenic [41] or genetically-related PSC lines. The use of multiple lines in this way adds confidence in the robustness of the observations made and their applicability to our understanding of the disease.

## Methods

### PSC lines

The PSC lines used are detailed in **Supplementary Table 1, Additional file** **1**. They include an isogenic series of ESCs, termed the IsoHD lines, comprising lines expressing wild-type (30 HTT polyglutamine (Q) repeats) or mutant (45 or 81 polyQ) HTT, generated and validated as described in Ooi et al [41]. Other lines are iPSCs derived at the UCL Queen Square Institute of Neurology. They were generated from fibroblasts in skin biopsies taken from, in the case of the HD family series, three siblings with juvenile Huntington’s disease, carrying *HTT* mutations with 56, 67 and 73 CAG repeats respectively, and their unaffected parent (20 CAG repeats) as a non-disease control; unrelated lines expressing HTT with 125 or 15 polyglutamine repeats were derived in the same manner. HTT polyglutamine lengths are expressed as *HTT* CAG(n)-length plus two (CAA, CAG) alleles; lengths below 36 repeats correspond to wild-type HTT, whilst those above correspond to the mutant form of the protein. 

For the iPSCs, briefly, biopsies were performed in accordance with the Declaration of Helsinki and approved by the University College London (UCL)/UCL Hospitals Joint Research Ethics Committee (LREC 03/N008, amendment 16). The subjects were recruited through the HD clinic at the National Hospital for Neurology and Neurosurgery, London; all subjects provided informed written consent. Two adjacent 3 mm punch biopsies taken from each subject’s forearm were cut into squares of 0.5-1 mm^2^ and placed epidermis side up into a well of a 6-well plate containing two drops of pre-warmed fibroblast culture medium (DMEM with glutamax, 4.5 g/L glucose and 1 mM pyruvate (Gibco, Thermo Fisher Scientific), 10% foetal bovine serum, 50 U/ml penicillin, 50 μg/ml streptomycin, 2.5 ml/L amphotericin). A sterile coverslip was placed over the pieces of tissue to aid adherence to the plate and a further 2 ml pre-warmed medium was added to the well. Following incubation for one week at 37 °C, 5% CO_2_, the media was changed; henceforth, cells were media changed every 3–4 days and passaged when required using 0.05% Trypsin-EDTA, reseeding each time at 1 × 10^4^ cells/cm^2^. During the expansion of the cultures, cells were frozen for storage in liquid nitrogen. Induced PSCs were generated by Sendai virus reprogramming of the fibroblasts using the CytoTune-iPS 2.0 Sendai reprogramming kit (Thermo Fisher Scientific). These were validated by the expression of pluripotency markers, differentiation into all germ layers using a self-organisation assay, karyotyping, Sanger sequencing to confirm the *HTT* CAG repeat length and confirmation of the absence of exogenous Sendai virus; two or three validated iPSC clones were generated from each subject. All PSCs were cultured routinely using Essential 8 medium on surfaces coated with Geltrex LDEV-free, hESC-qualified, reduced growth factor basement membrane matrix, both used according to the manufacturer’s instructions (Gibco, Thermo Fisher Scientific).

### Microglia differentiation

Microglia were differentiated according to published methods [42, 43]. Briefly, embryoid bodies were formed by seeding 10,000 PSCs/well in round-bottomed ultra-low attachment 96-well plates, using Essential 8 medium supplemented with 50 ng/ml human BMP4 (Peprotech), 50 ng /ml human VEGF (Cell Guidance Systems), 20 ng/ml SCF (Cell Guidance Systems) and 10 mM Y27632 ROCK inhibitor (Sigma-Aldrich, Merck). After five days, the embryoid bodies were collected and transferred at one per cm^2^ in flasks containing X-Vivo 15 medium (Lonza) with 2 mM l-glutamine, 100 ng/ml human M-CSF (Cell Guidance Systems), 25 ng/ml IL-3 (Cell Guidance Systems) and 50 μM 2-mercaptoethanol. After three weeks, tissue-resident macrophage precursors were first harvested and at weekly intervals thereafter. These cells were cultured in Corning Primaria plates using DMEM/F12 medium (Gibco, Thermo Fisher Scientific) supplemented with 2 mM l-glutamine, 100 ng/ml GM-CSF (Cell Guidance Systems), 100 ng/ml human IL-34 (R&D Systems, Biotechne), and 50 units/ml penicillin and 50 μg/ml streptomycin, to promote the differentiating cells towards a mature microglial state. They were compared with primary human monocytes and macrophages obtained and cultured as described previously [27].

### Striatal neuron differentiation

Cells were differentiated to a striatal neuron fate using a published method [44]. Briefly, PSC monolayers were first directed to a forebrain neural progenitor fate by way of the presence of N2 and B27 supplements (Gibco, Thermo Fisher Scientific) and BMP and SMAD inhibitors (100 nM LDN193189 (Sigma-Aldrich, Merck); 200 nM dorsomorphin (Cambridge Biosciences); 10 μM SB431542 (Cambridge Biosciences)) over a 9-10 day period. Thereafter, patterning to a lateral ganglionic eminence/striatal fate was achieved by the addition of 25 ng/ml activin A (PeproTech), which was maintained throughout the remainder of the differentiation process. The addition of 10 ng/ml human BDNF (PeproTech), 10 ng/ml human GDNF (PeproTech) and vitamin A-containing B27 for 10 days from day 26 post-induction onwards aided maturation of these striatal precursors to a more mature striatal neuron identity, resulting in cultures expressing markers of striatal GABAergic neurons, including a proportion expressing a marker of full maturity, DARPP32.

### Treatments of cell cultures

PSC-derived microglia were stimulated with 1 μg/ml LPS (O55:B5 *E. coli;* Sigma-Aldrich, Merck) and 10 ng/ml human IFNγ (R&D Systems, Biotechne) for periods of time appropriate to individual experiments, as indicated in the relevant figure legends. They were stimulated with 110 μM tert-butyl hydroperoxide (TBHP) in 10% foetal bovine serum for four hours to induce ROS release. To induce stress, they were treated with either 20–40 ng/ml bafilomycin A1 or 8–12 mM hydrogen peroxide (H_2_O_2_), as described in the relevant figure legends. They were treated with human cerebrospinal fluid (CSF), obtained from the HD-CSF study [45] and diluted 1:2 in the appropriate media, for 24 h. For the conditioned media experiments, PSC-derived microglia and striatal neurons were cultured in conditioned media from the other cell type diluted 1:2 in their normal culture media.

### Quantitative PCR

RNA was isolated using Qiagen’s RNeasy Mini Kit Plus kit, following the manufacturer’s instructions. cDNA conversion was undertaken using Invitrogen Superscript III reverse transcriptase (Thermo Fisher Scientific), also as per the manufacturer’s instructions; qPCR was conducted using Applied Biosystems’ SYBR-Green PCR Master Mix and gene-specific oligonucleotide primers, including those for endogenous reference genes (*ACTB* and *GAPDH*) (sequences are provided in **Supplementary Table 2, Additional file** **1**). Three parallel reactions were conducted as technical replicates per single data point. The ΔΔCt method was applied to report the geometric mean of relative fold-changes of data normalised to that of each reference gene.

### High-content immunofluorescence imaging

Cells were fixed with 10% formalin solution (containing 4% paraformaldehyde; Sigma-Aldrich, Merck) for 15 min and washed three times with PBS prior to permeabilisation with 0.2% Triton X-100 in PBS for 15 min, both steps at room temperature. Blocking solution containing 10% donkey or goat serum and 10% BSA in PBS was applied for 1 h at room temperature. Primary antibodies (details are provided in **Supplementary Table 3, Additional file** **1**) were added in 1% BSA in PBS and incubated overnight at 4 °C. After five gentle washes with PBS, secondary antibodies were added at a dilution of 1:1000 and incubated for 1 h in the dark at room temperature. After two further PBS wash steps, 1 μg/ml Hoechst 33342 in PBS was added for 5 min at room temperature. The stained cells were washed twice more with PBS, prior to addition of 0.02% sodium azide in PBS for storage and imaging. Secondary antibody-only controls were used to verify staining was specific.

High-content images were captured using the Perkin Elmer Opera Phenix HCS system, with twenty pre-set fields-of-view captured per well using a ×40 air NA 0.6 objective. Six technical replicate wells were imaged and analysed per data point. Channels for Hoechst, and 488, 568 and 647 nm were selected, the power set to 100% and separated where appropriate to prevent crosstalk. The plane and gain settings were kept constant for each channel and each antibody combination. Images were analysed using Columbus image analysis software. In summary, individual planes were analysed (without flatfield correction), as follows: Nuclei were identified and categorised by means of the intensity and area of Hoechst staining and the software’s Find Nuclei function—these parameters were kept constant for each subsequent algorithm; βIII-tubulin+, MAP2+ and activated caspase 3+ cells were detected using the Find Cytoplasm function; DARPP32+ and nestin+ cells were identified using the Find Cells function; CTIP2+ cells were determined by thresholding of staining in the nucleus; and nuclear Ki67 and S830 puncta were identified using the Find Spots function (a full description is provided in **Supplementary Table 4, Additional file** **1**). Appropriate population and formula outputs were defined, downloaded and exported for statistical analysis.

### Cytokine multiplex ELISA

Cytokine profiling was carried out using MesoScale Discovery V-PLEX Proinflammatory Cytokine Panel 1 (Human) ELISA kits, following the manufacturer’s instructions. Supernatants from LPS-stimulated and unstimulated cultures were diluted 1:300 and 1:5, respectively. Three parallel wells as technical replicates were measured per single data point. Where possible, data were normalised to total cell number as measured by high-content imaging. Otherwise, they were normalised to total cell protein content as measured by Pierce BCA assay (Thermo Fisher Scientific), having been washed once with sterile D-PBS and lysed in 60 μl RIPA buffer supplemented with Roche cOmplete Mini EDTA-free protease inhibitor cocktail.

### Reactive oxygen species and nitric oxide assays

Reactive oxygen species (ROS) were assayed using Abcam’s DCFDA/H2DCFDA assay kit (Abcam), used according to the manufacturer’s instructions. Cells were seeded at 25,000 cells/well in Perkin Elmer CellCarrier 96-well plates for use in the assay; data were obtained by measuring fluorescence at 485/535 nm (excitation/emission) using a Tecan Spark microplate reader at specific time-points according to the design of each experiment. Three parallel wells as technical replicates were measured per single data point.

Nitrite and nitrate levels were assessed either using Abcam’s fluorometric nitric oxide assay kit, following the manufacturer’s instructions, or in the case of nitrite levels in supernatants specifically, by the Greiss method [46]. The latter required a comparison of the 540 nm absorbance levels of 200 μl supernatants or standards containing 0–100 μM nitrite to which 100 μl Greiss reagent (0.1 % naphthylethylenediamine, 1 % sulphanilamide, 5 % H_3_PO_4_) had been added.

### Phagocytosis assay

Cells were seeded for maturation at 100,000 cells/well in Perkin Elmer Cell Carrier-96 plates. Cultures were then washed three times with D-PBS prior to incubation with HBSS (with Ca^2+^ and Mg^2+^) and 20 mM HEPES for 1 h. Sonicated pHrodo Zymosan A green or *Escherichia coli* red fluorescent BioParticles (Thermo Fisher Scientific) were applied to the cultures for 2–3 h for maximal phagocytosis. Six parallel wells as technical replicates were measured per single data point. Fluorescence was measured using a Tecan Spark microplate reader set at excitation/emission 509/533 nm for zymosan green and 560/585 nm for *E. coli* red particles, respectively.

### Assays of cell viability

Cell viability was analysed using Promega’s CytoTox 96 Non-Radioactive Cytotoxicity Assay kit, following the manufacturer’s instructions. In so doing, maximal LDH release controls were performed for each well to allow interpretation of the level of LDH as a percentage of total LDH to control for cell number. Apoptosis was assessed using Promega’s RealTime-Glo Annexin V Apoptosis Assay kit, according to the manufacturer’s instructions. In both cases, three parallel wells as technical replicates were measured per single data point.

### Statistics

Data were analysed as the mean of at least three differentiations of each of the IsoHD ESC lines or at least one differentiation of each of three clones of the HD family iPSC lines, unless indicated otherwise. Where multiple lines were compared with each other, ostensibly to identify effects of the presence of mutant HTT, the data were subject to one-way ANOVA with Tukey’s post-test for multiple comparisons. Where the two series of lines, that is the IsoHD and the HD family series, were compared with each other, the data were analysed by unpaired Student’s *t*-test. Where lines were differentiated to one cell type and received conditioned supernatants from the same lines differentiated to another, again ostensibly to identify effects of the presence of mutant HTT, the data were subjected to two-way ANOVA with Tukey’s post-test for multiple comparisons. Where multiple lines of varying degrees of relatedness were compared with the aim of identifying effects that might be HTT polyglutamine length dependent, that is showing some relationship that spans the range of both pathogenic and non-pathogenic HTT polyglutamine repeats, data were analysed by linear regression in which slopes were determined to be non-zero or not. The overall effect of any given treatment, such as activation of microglia with LPS or subjecting cultures to a stress, was assessed by paired Student’s *t*-test. Where multiple lines on the same genetic background were subject to a treatment over a time course, data were analysed by linear regression analysis in which slopes were compared with each other. A 95% confidence interval (*p* < 0.05) was considered a statistically significant observation. All statistical analyses were conducted using GraphPad Prism 6.07.

## Results

### Huntington’s disease PSCs show no deficits in their ability to differentiate to mature microglia

PSC lines of various HTT polyglutamine lengths were differentiated to a microglial fate by inducing tissue macrophage-like characteristics in cells of a myeloid lineage [42]. This included lines of an isogenic series of ESCs, termed the IsoHD series, comprising lines with 30, 45 and 81 HTT polyglutamine repeats [41], and lines belonging to an iPSC series, termed the HD family series, comprising lines of 22, 58, 69 and 75 polyglutamine repeats. Other, unrelated iPSC lines expressing HTT with 15, 20 and 125 polyglutamine repeats were also examined. Using this method, each of the PSC lines used in the study generated CD14+/TREM2+/IBA1+/TMEM119+/PU1+ cells that are confirmative of mature microglia. To assess potential HTT polyglutamine length-dependent effects on differentiation and/or cell viability, total cell numbers at the end of the differentiation process were quantified. This showed no mutant HTT-dependent effects on total cell number in either the IsoHD or the HD family series of related lines (**Supplementary Fig. 1a, Additional file** **2**). As might be expected, therefore, linear regression analysis of all the lines together showed no HTT polyglutamine length-dependent effects on total cell number (*r*^2^ = 0.0652; **Supplementary Fig. 1b, Additional file** **2**). Taking all the lines within a series together, however, showed that the lines of the IsoHD series consistently generated higher total cell numbers than those of the HD family series, plausibly demonstrating an effect of genetic background on the yields of cells obtained when using this differentiation protocol (*p* = 0.024; **Supplementary Fig. 1c, Additional file** **2**).

**Fig. 1:**
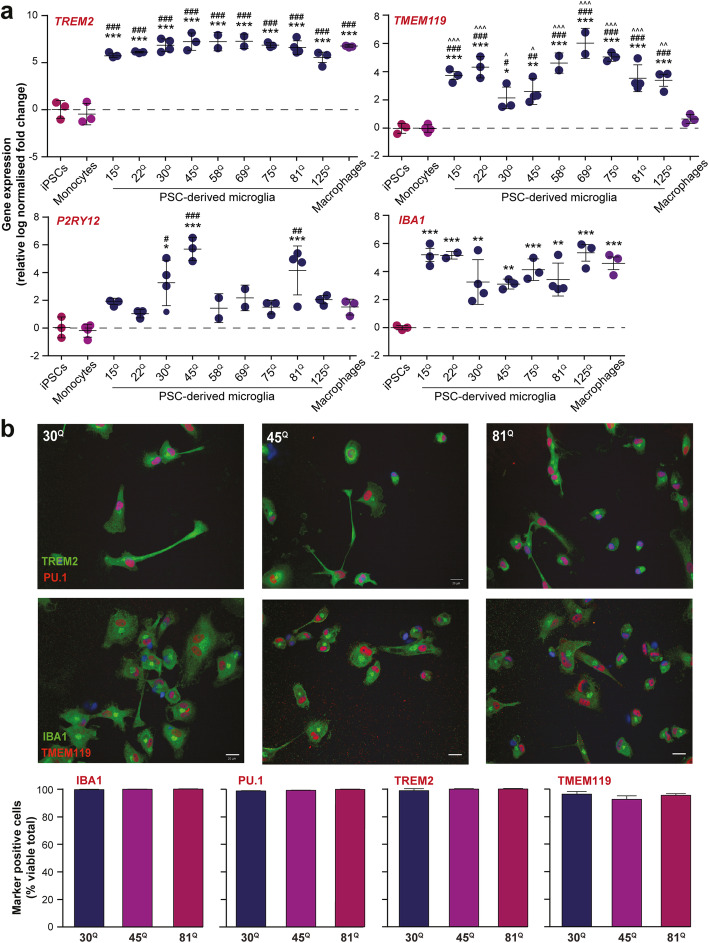
Mutant HTT does not affect the differentiation of human PSC-derived microglia**. a** PSC lines expressing HTT of different polyglutamine repeat lengths were differentiated to a microglial fate. They were assessed for the expression of genes associated with microglial identity, both to confirm generally the robustness of the methods used across multiple lines and to test specifically for any mutant HTT-dependent effects on differentiation. The macrophage markers, *TREM2* and *IBA1*, were significantly elevated in each of the lines tested compared with iPSCs and, for the former at least, primary human monocytes. *TMEM119*, a gene whose expression is associated with microglia/tissue-resident macrophages when compared with their systemic, monocyte-derived counterparts, was elevated in each of the lines tested compared with iPSCs, primary monocytes and monocyte-derived macrophages. *P2RY12,* a gene whose expression also is associated with microglia/tissue-resident macrophages, was far more variable in its expression yet was significantly elevated in some of the lines. Taken together, these data indicate the robustness of the methods used across all the lines, but do not show any mutant HTT-dependent effects on differentiation. **b** IsoHD PSC lines expressing HTT of different polyglutamine repeat lengths were differentiated multiple times to a microglial fate. Quantification of the numbers of cells expressing protein markers of microglial identity was undertaken by high-content immunofluorescence microscopy. This showed that the cultures were almost entirely enriched for microglial-like cells expressing TREM2, IBA1, TMEM119 and PU1. There were no HTT polyglutamine repeat-dependent differences in proportions of cells that were positive for these protein markers. Data are presented as mean ± SEM, analysed by one-way ANOVA with Tukey’s multi-comparison post-test (*/#/^ *p* < 0.05, **/##/^^ *p* < 0.01, ***/###/^^^ *p* < 0.001; where * = vs iPSCs, # = vs primary monocytes, ^ = vs monocyte-derived macrophages)

Following differentiation, putative PSC-derived microglia were assessed by qPCR for expression of gene markers indicative of a macrophage fate, or indeed a tissue-resident macrophage fate specifically, as compared with undifferentiated PSCs, primary human monocytes and monocyte-derived macrophages. Each of the PSC lines expressed the macrophage markers, *TREM2* and *IBA1*, with significantly elevated transcript levels compared with those of undifferentiated iPSCs and primary monocytes (Fig. 1a). They also expressed the tissue-resident macrophage/microglia marker, *TMEM119*, at elevated levels compared with those of undifferentiated iPSCs, primary monocytes and monocyte-derived macrophages, as expected (Fig. 1a). Another marker of tissue-resident macrophages/microglia, *P2RY12*, showed considerably more variation in transcript expression between the PSC-derived microglial cultures, yet expression was still significantly elevated in at least some of the lines compared with both undifferentiated iPSCs and primary monocytes (Fig. 1a). Other genes that would be expected to express at higher levels in tissue-resident macrophages/microglia as compared with monocytes, *C1QA*, *GAS6*, *GPR34*, *MERTK* and *PROS1*, were also each elevated as expected in each of the lines that were tested (**Supplementary Fig. 2a, Additional file** **2**). Similarly, *CD14* and *CD93*, genes that would be expected to be expressed at lower levels in tissue-resident macrophages/microglia compared with myeloid cells of monocyte origin, did indeed show lower expression in PSC-derived microglia compared with primary monocytes (**Supplementary Fig. 2b, Additional file** **2**). Finally, *NANOG*, *POUF1*, *SOX2* and *ZFP42*, genes whose expression is associated with the pluripotency of PSCs were significantly downregulated in microglia compared with the PSCs they were derived from (**Supplementary Fig. 3, Additional file** **2**).

Taken together, these data demonstrate consistency in the microglial-like identity, as opposed to a more monocyte-like one, of cultures differentiated from multiple PSC lines, irrespective of their origin or how they were derived. There was some apparent, although not statistically significant, variability between lines in the degree to which the genes tested was up- or downregulated in the expected manner; this was most pronounced for the expression of *P2RY12*, which appeared more highly expressed in the IsoHD lines than the others (Fig. 1a). Indeed, when the lines of a series were taken together and the series compared, the IsoHD series collectively did have a significantly higher *P2RY12* expression than the HD family series (*p* = 0.0075), possibly demonstrating effects of genetic background and/or an altered susceptibility to the culture environment on the expression of this gene in the differentiated cells. It is this variability, perhaps, that points to why the apparently elevated expression of *P2RY12* lines of the HD family series compared with that of undifferentiated iPSCs or primary monocytes was not of statistical significance. Moreover, it may be the case that upregulated levels of P2RY12 protein do not show the same variability in expression as observed for the gene.

Importantly, however, there were no significant differences between lines expressing mutant HTT and those that do not. Similarly, linear regression analysis of all the lines together showed no HTT polyglutamine length-dependent effects on the expression levels of any of the genes tested, with the exception of *MERTK*, which showed some relationship between increasing HTT polyglutamine length and elevated gene expression (*r*^2^ = 0.3467; *p* = 0.0267). Taken together, therefore, the data show little effect of HTT polyglutamine length on the differentiation of human PSC-derived microglia and, more importantly, no evidence that mutant HTT per se impairs the differentiation of microglia from PSCs.

To assess the purity of the microglia cultures differentiated from human PSCs, cultures were subject to high-content immunofluorescence microscopy of the markers TREM2, IBA1, TMEM119 and PU.1. Quantification of the numbers of cells expressing these proteins showed that almost all were positive for all four markers of microglial fate, demonstrating the robustness of the method used and the homogeneity of the cultures produced (Fig. 1b). There were no differences in the proportions of cells expressing these markers, or the immunofluorescence staining intensity of each marker (**Supplementary Fig. 4, Additional file** **2**), between lines expressing mutant HTT and those that do not, confirming that mutant HTT does not impact microglial development, at least in this in vitro context.

### Huntington’s disease PSC-derived microglia produce elevated levels of cytokines in response to an immune challenge

**Fig. 2:**
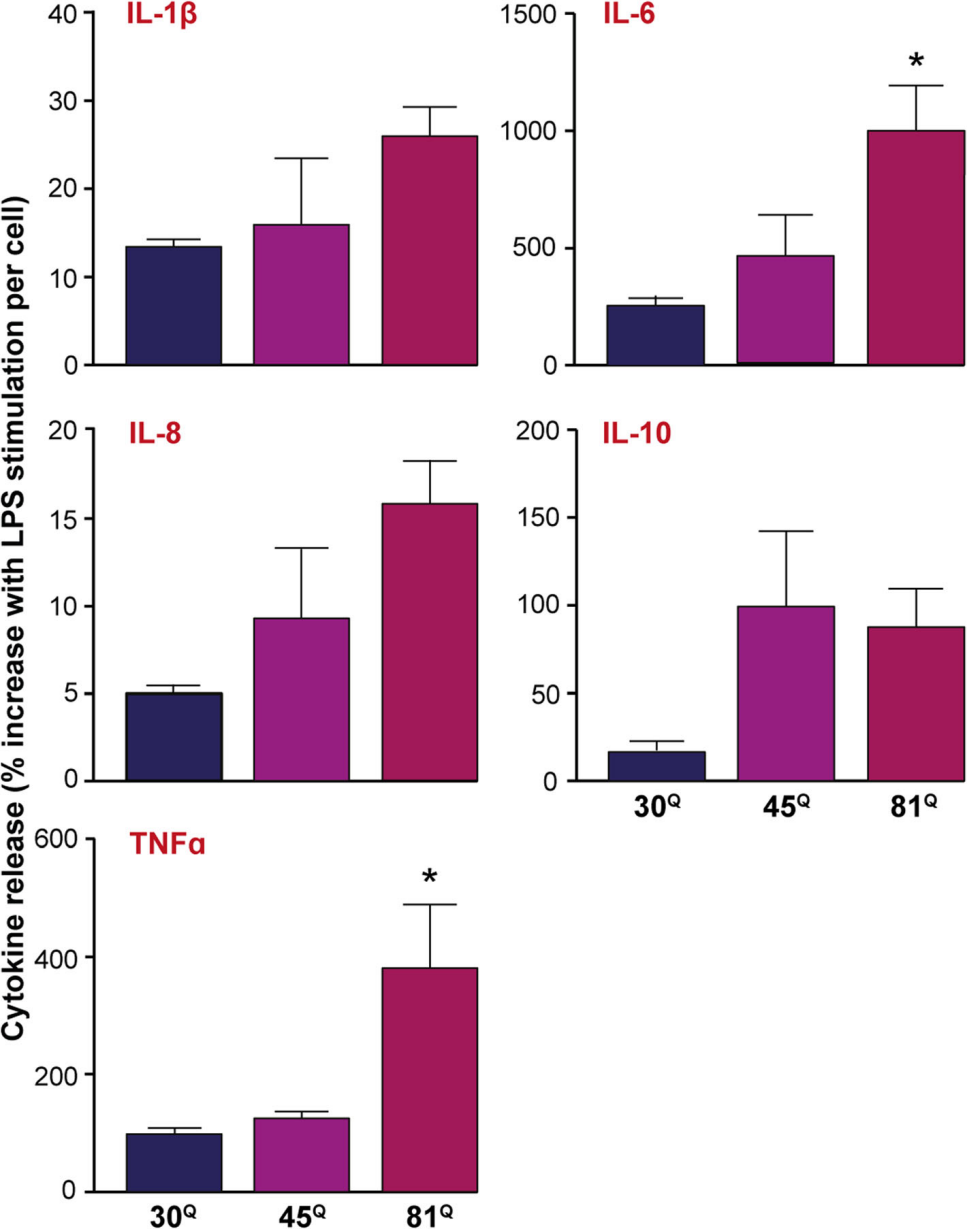
Human Huntington’s disease PSC-derived microglia are hyper-reactive to an immune challenge. **I**soHD PSC-derived microglia were stimulated with 1 μg/ml LPS and 10 ng/ml IFNγ for 24 h and their supernatants assessed by multiplex ELISA for the secretion of inflammation-associated cytokines. In most cases, there was a trend of increased production of the cytokines measured, with statistically significantly elevated levels of the pro-inflammatory cytokines, IL-6 and TNFα. Data are expressed as the fold-change following stimulation normalised to total cell number, presented as mean ± SEM, *n* = 3, and analysed by one-way ANOVA with Tukey’s post-test, (* *p* < 0.05)

It has been shown previously that peripheral HD myeloid cells (i.e. monocytes and their derivatives) of human origin release higher levels of cytokines associated with inflammatory responses when stimulated with an activating challenge such as LPS [6, 27]. Likely this hyper-reactive phenotype explains the elevated levels of these cytokines in the plasma of HD gene carriers. That human microglia might behave in the same manner has been hypothesised but not demonstrated to date. Here, IsoHD PSC-derived microglia were stimulated with LPS and their supernatants assessed for levels of inflammation-associated cytokines. This showed that each of the pro-inflammatory cytokines tested appeared elevated in a HTT polyglutamine length-dependent manner. More specifically, IL-6 and TNFα were produced at increased levels by mutant HTT-expressing microglia (*p < 0.05, 30^Q^ vs 81^Q^; Fig. 2), akin to observations made in their primary human monocyte counterparts. In contrast, HTT polyglutamine-dependent effects on phagocytosis were not observed (**Supplementary Fig. 5, Additional file** **2**). This suggests that autonomously hyper-responsive microglia are indeed a likely constituent part of the CNS cellular milieu where HD pathology is most prominent.

### Huntington’s disease PSC-derived microglia produce elevated levels of reactive oxygen species

**Fig. 3:**
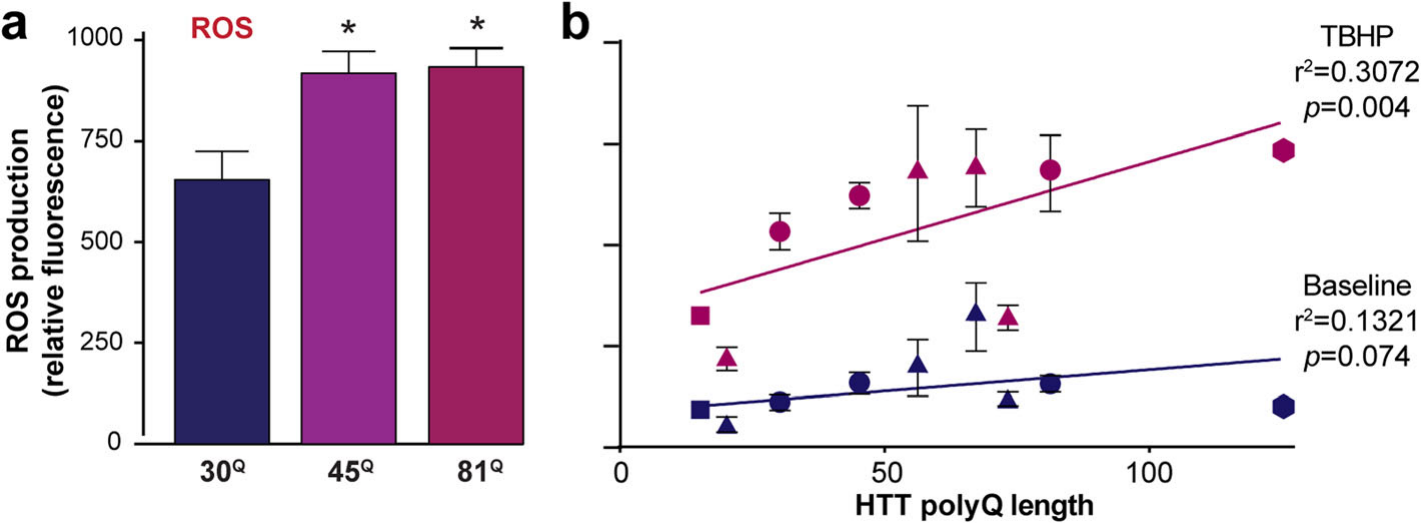
Human Huntington’s disease PSC-derived microglia show elevated production of reactive oxygen species. **a** IsoHD PSC-derived microglia were assessed by DCFDA/H2DCFDA assay for the steady-state production of ROS. This showed statistically significantly increased levels of ROS production by microglia expressing HTT with pathogenic polyglutamine repeats compared with the non-pathogenic, 30^Q^ control. Data are presented as mean ± SEM, *n* = 3, analysed by one-way ANOVA with Tukey’s post-test (*p < 0.05). **b** IsoHD and other PSC-derived microglia were then assessed for production of ROS with and without treatment with 110 μM TBHP, a ROS-inducing toxin, for 4 h. Under non-treated, baseline conditions, linear regression analysis of all the lines did not show a significant HTT polyglutamine-dependent effect on ROS production. However, under the TBHP-treated condition, a HTT polyglutamine-dependent effect was observed, as shown by a linear regression slope that was significantly non-zero. Data are presented as mean, ± SEM where multiple measurements of the same line were made. The identity of the lines used are as follows: circles = lines of the IsoHD ESC series, triangles = lines of the HD family iPSC series, square = 1534 iPSC line, hexagon = QS5.1 line

Neuroinflammation is typically associated with the microglial release of ROS that directly causes damage to neurons by way of oxidative effects on their nucleic acids, proteins and lipids. Here, supernatants from IsoHD PSC-derived microglia were assessed for their levels of ROS production. This showed that significantly elevated levels of reactive oxygen species were released by microglia expressing HTT with pathogenic polyglutamine repeats (*p<0.05, 30^Q^ vs 45^Q^ and 30^Q^ vs 81^Q^; Fig. 3a). When microglia of both the IsoHD and other PSC lines were then assessed together for the production of ROS with and without treatment with a ROS-inducing toxin, TBHP, linear regression analysis suggests a significant HTT polyglutamine-dependent effect on ROS production under the TBHP-treated condition (*r*^2^ = 0.3072, *p* = 0.01; Fig. 3b), although the relationship is only partial and the larger effect may be a categorical difference between pathogenic and non-pathogenic HTT polyglutamine repeats rather than a strict linear relationship. Still, these data demonstrate that the hyper-responsive phenotype of human HD microglia extends to the production of molecules known to be neurotoxic in a pathogenic context. In contrast to the differences observed in ROS levels, however, no mutant HTT-dependent differences in the production of reactive nitrogen species were observed (**Supplementary Fig. 6, Additional file** **2**).

### Huntington’s disease PSC-derived microglia are more vulnerable to exogenous stress

**Fig. 4:**
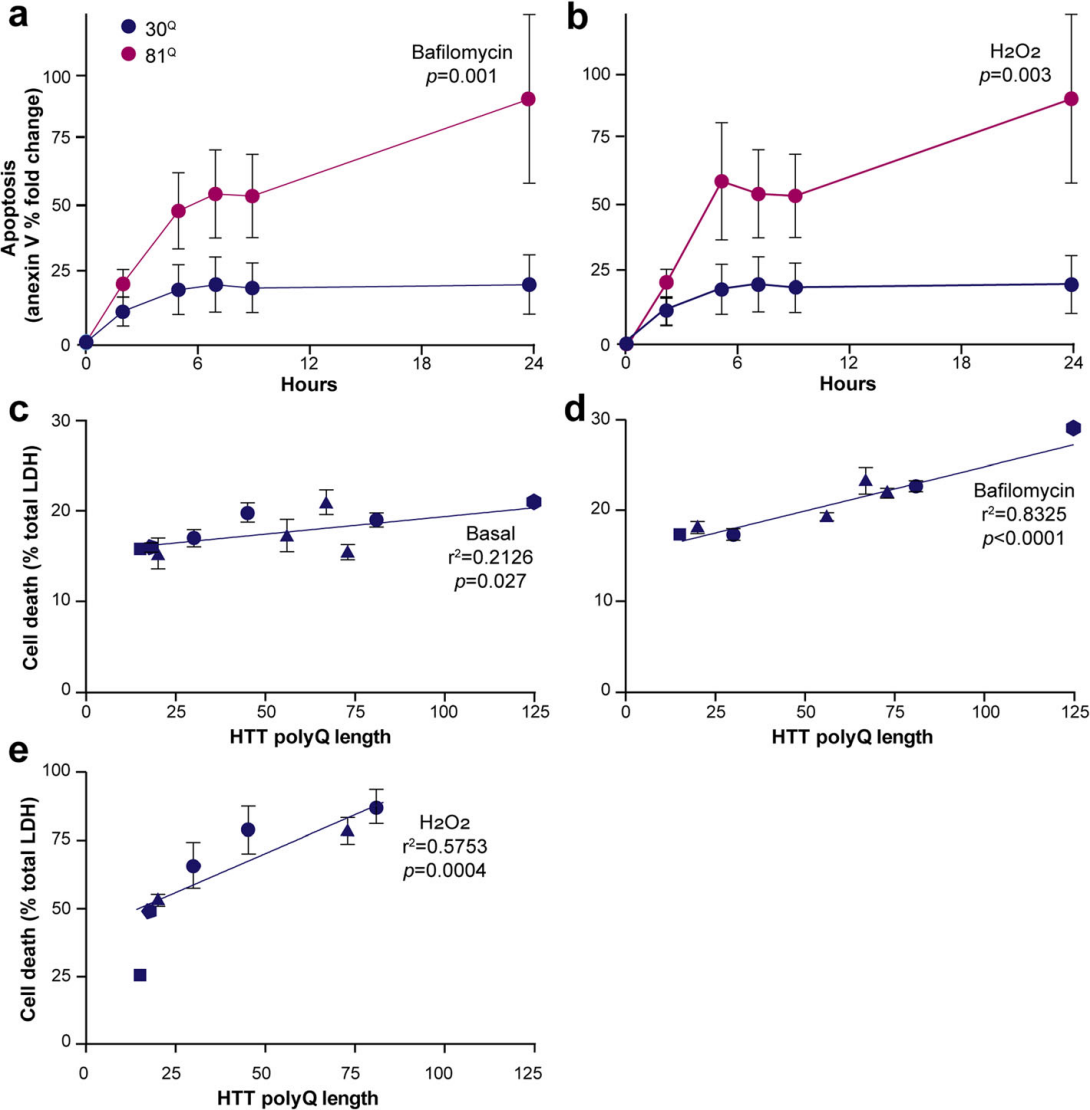
Human Huntington’s disease PSC-derived microglia show increased levels of apoptosis and are more susceptible to exogenous stress. **a**, **b** IsoHD PSC-derived microglia were assessed longitudinally up to 24 hours for annexin V binding as a marker for apoptosis in response to stress. **a** Exposure to 40 ng/ml bafilomycin A1 resulted in elevated apoptosis over time in HTT 81^Q^ cells compared with 30^Q^ ones as shown by a significantly non-zero linear regression slope. **b** Similarly, exposure to 8 mM H_2_O_2_ also showed elevated apoptosis over time in the HTT 81^Q^ expressing cells by a significantly non-zero linear regression slope. **c–e** IsoHD and other PSC-derived microglia were assessed for LDH release as a measure of overt cell death under **c** basal conditions and in response to the stress caused by treatment with **d** 20 ng/ml bafilomycin A1 for nine hours and **e** 12 mM H_2_O_2_ for one hour. In each case, linear regression analysis of the relationship between HTT polyglutamine-length and percentage cell death showed a significantly non-zero slope, with the slopes being steeper following stress than at baseline. Data are presented in **a** and **b** as mean, ± SEM, *n* = 3. Data are presented in **c**–**e** as mean, ± SEM where multiple measurements of the same line were made. The identity of the lines used in **c**–**e** are as follows: circles = lines of the IsoHD ESC series, triangles = lines of the HD family iPSC series, square = 1534 iPSC line, hexagon = QS5.1 line

The environment of the HD brain likely becomes progressively toxic as neural cells become increasingly compromised. It is also possible that mutant HTT-expressing microglia are intrinsically more vulnerable to exogenous sources of cellular stress, as might be expected of a neuropathological environment. To determine whether the resilience of human PSC-derived microglia is affected by the expression of mutant HTT, their viability was assessed in response to various forms of cellular stress. When exposed to bafilomycin A1, an autophagy inhibitor known to cause cell stress [47], at levels sufficient to cause a mild loss of viability overall (*p* = 0.035), levels of apoptosis showed a significant mutant HTT-dependent effect on annexin V binding (*p* = 0.001, 30^Q^ vs 81^Q^; Fig. 4a) and a HTT polyglutamine length-dependent effect on LDH release (*r*^2^ = 0.8325, *p* < 0.0001; Fig. 4d). When exposed to hydrogen peroxide, a more overt challenge to the cells such that oxidative stress causes a substantial loss of viability overall is observed, the effects were similar, with a significant mutant HTT-dependent effect on annexin V binding (*p* = 0.003, 30^Q^ vs 81^Q^; Fig. 4b) and a HTT polyglutamine length-dependent effect on LDH release (r^2^ = 0.5753, *p* = 0.0004; Fig. 4e). Under basal conditions, levels of apoptosis also showed some dependence on HTT polyglutamine length, although the effect appeared less overt (*r*^2^ = 0.2126, *p* = 0.027; Fig. 4c). Taken together, these data suggest that modest yet significantly elevated basal levels of apoptosis in HD PSC-derived microglia become more pronounced when the cells are exposed to exogenous stress.

### Conditioned media from human Huntington’s disease striatal neurons elevate apoptosis in human PSC-derived microglia undergoing cellular stress

**Fig. 5:**
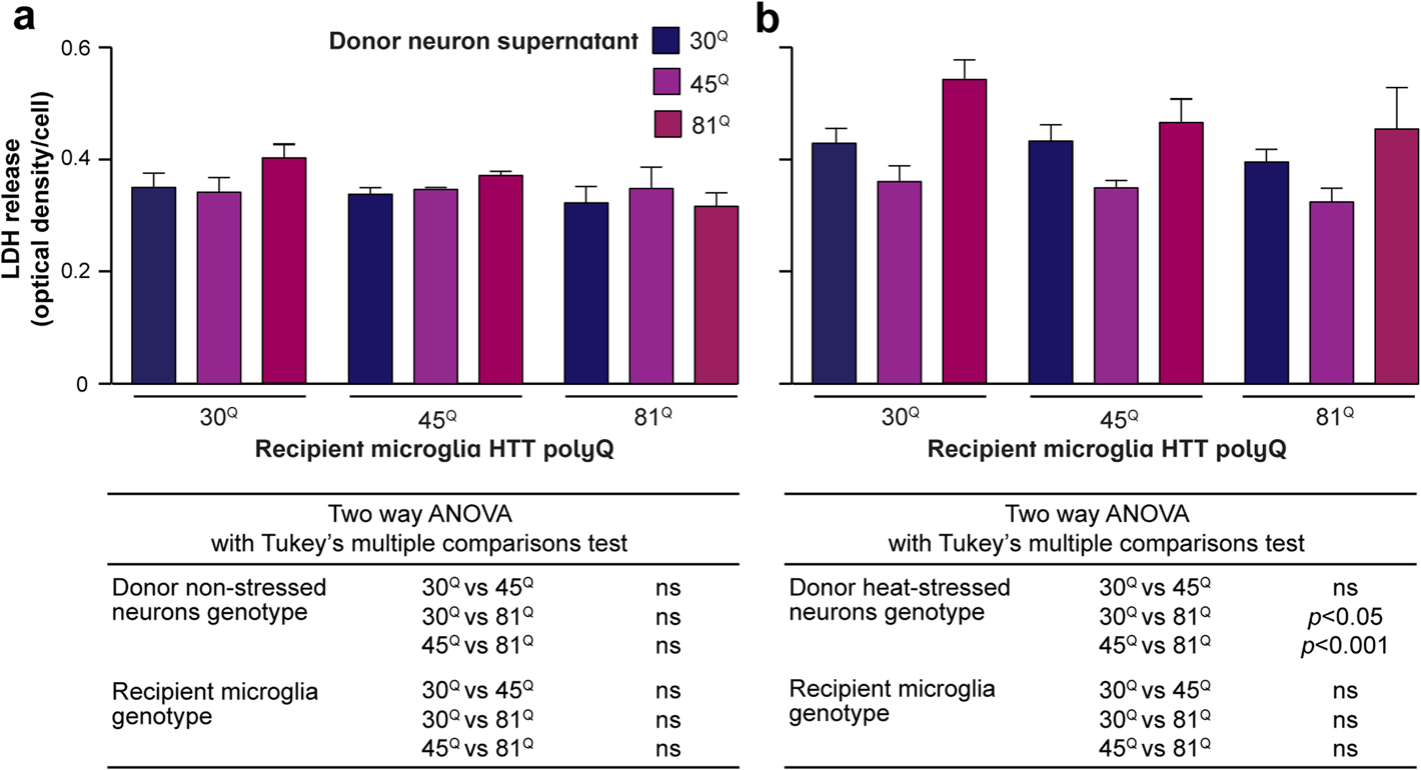
Human PSC-derived microglia show elevated cell death when treated with media from human PSC-derived striatal neurons expressing mutant HTT with a long polyglutamine-tract. IsoHD PSC-derived microglia were cultured with supernatants harvested from IsoHD PSC-derived striatal neurons. **a** Exposure to supernatants from untreated neuronal cultures for five days did not reveal any HTT polyglutamine-dependent effects, either of the donor neurons or the recipient microglia, on LDH release by the microglia. **b** By contrast, when the neurons had been heat-shocked at 45 °C for 45 min to induce stress, recipient microglia of all HTT genotypes showed elevated LDH release when treated with supernatants from HTT 80^Q^ neurons compared with those from the 30^Q^ and 45^Q^ ones. Data are presented as mean ± SEM, *n* = 3, analysed by two-way ANOVA with Tukey’s multiple comparisons test

Given the apparent susceptibility of human HD PSC-derived microglia to exogenous stress, we sought to determine whether stress factors specifically associated with HD pathology could exert a similar effect. Striatal GABAergic medium spiny neurons are selectively vulnerable in HD. Such cells can be derived from human PSCs [44], including those carrying the HD mutation. Here, striatal neurons were differentiated from each of the IsoHD lines, expressing HTT with either non-pathogenic (30^Q^) or pathogenic (45^Q^ and 81^Q^) polyglutamine repeat lengths (**Supplementary Fig. 7, Additional file** **2**). Their supernatants were harvested and used to treat cultures of microglia derived from the same selection of lines. Following treatment for five days, no increases in microglial cell death were observed (Fig. 5a). It may be that the PSC-derived striatal neurons used here are not affected sufficiently by the expression of mutant HTT with expanded polyglutamine tract lengths. Or it may be that these cells when in culture are not necessarily exposed to a significant extent to the full secreted milieu associated with the degenerative environment of the disease. Under such conditions, it is entirely likely that neurons expressing mutant HTT are more vulnerable to the stressors released as pathology progresses. Here, then, the cultured IsoHD striatal neurons were differentiated and subjected to heat-shock stress. Their supernatants were collected and used to treat PSC-derived microglia, as before. Subsequent measurements showed that overall levels of cell death in PSC-derived microglia were increased when treated with supernatants from striatal neurons that had been stressed compared with those that had not (*p* = 0.0085). Moreover, supernatants from striatal neurons with the longest HTT polyglutamine-length induced significantly higher levels of PSC-derived microglial cell death than those of the others (*p* < 0.05, 30^Q^ vs 81^Q^; *p* < 0.001, 45^Q^ vs 81^Q^; Fig. 5b). However, there were no microglial mutant HTT-dependent effects on their responses. Nonetheless, this suggests that a consequence of the effects of mutant HTT expression on the response to stress of neurons is a downstream effect on microglial viability.

To determine whether such observations could be mimicked when using a ‘real-world’ equivalent to the cell culture supernatants, namely cerebrospinal fluid (CSF) from HD patients, the experiments were repeated, but no differences were observed (**Supplementary Fig. 8, Additional file** **2**). This may be because CSF does not fully reflect the localised micro-environment in which cells find themselves within the tissue of the degenerating brain, or that the cultures require exposure either over a longer timescale and/or without the potentially insulating presence of cell culture media, neither of which is technically feasible, to observe an effect.

### Conditioned media from human Huntington’s disease microglia do not overtly affect the composition or viability of cultures of human PSC-derived striatal neurons

**Fig. 6.**
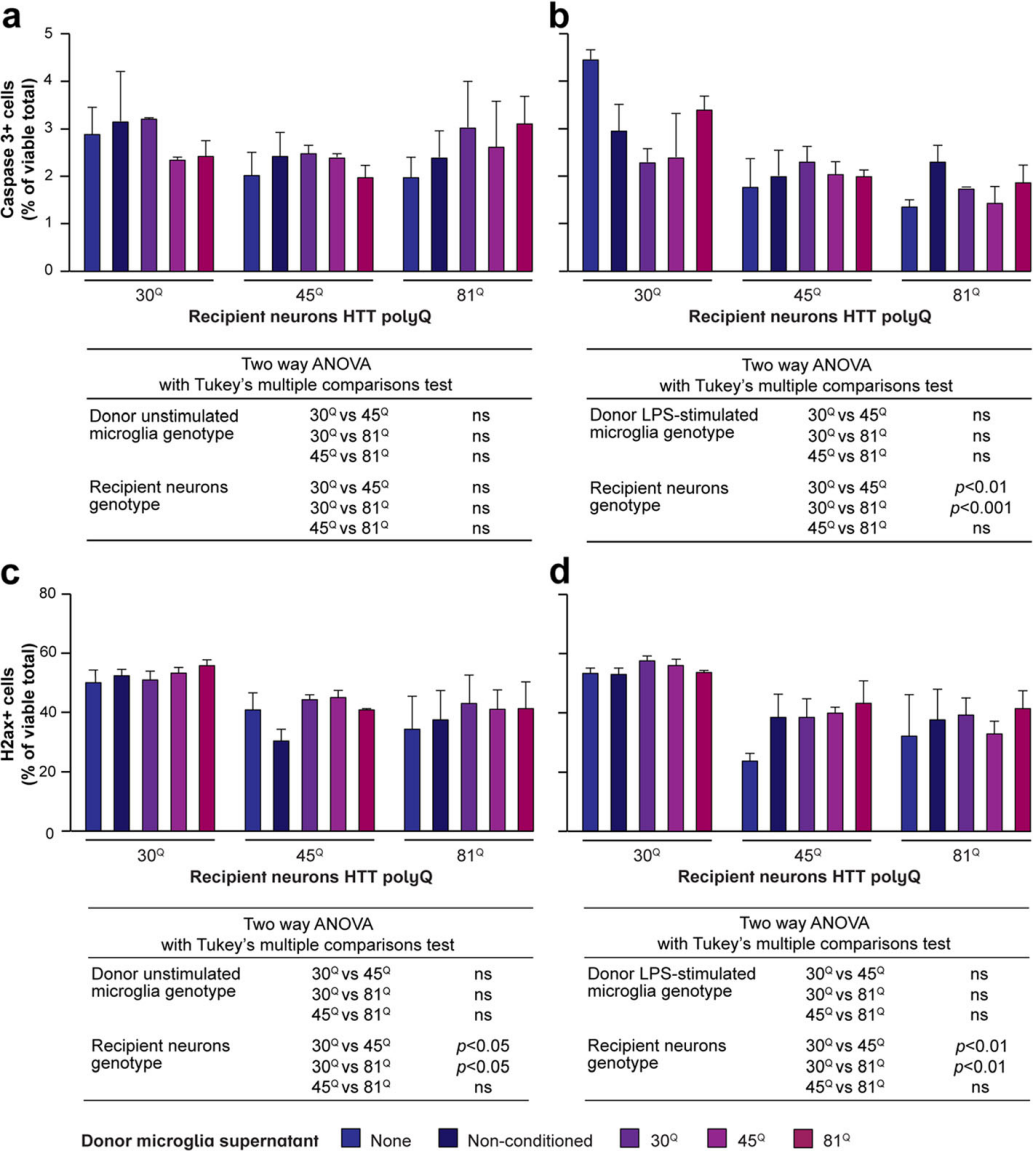
Human Huntington’s disease PSC-derived microglia conditioned media are not toxic to human PSC-derived striatal neurons. IsoHD PSC-derived striatal neurons were cultured with supernatants harvested from IsoHD PSC-derived microglia. **a**, **b** Numbers of recipient cells demonstrating caspase 3 activation as a marker of apoptosis were not affected by exposure for five days to conditioned media from microglia expressing mutant HTT, when either **a** the microglia were unstimulated or **b** the microglia had been activated by treatment with 1 μg/ml LPS and 10 ng/ml IFNγ for 24 h. **c**, **d** Numbers of recipient cells showing increased numbers of γH2ax foci, a marker of DNA damage and genotoxic stress, were lower in HTT 45^Q^ and 81^Q^ neurons than 30^Q^ ones, irrespective of whether they had been treated or not with conditioned media from either **c** unstimulated or **d** LPS-stimulated mutant HTT-expressing microglia. Data are presented as mean ± SEM, *n* = 3, analysed by two-way ANOVA with Tukey’s multiple comparisons test

Hyper-responsive HD microglia that secrete elevated levels of pro-inflammatory molecules might be expected to exert some detrimental effects on vulnerable neurons, particularly those that themselves express the disease-associated HTT mutation. Here, mature microglia were differentiated from each of the IsoHD lines and stimulated with LPS and IFNγ at levels sufficient to activate the cells in terms of pro-inflammatory cytokine release. Their supernatants were harvested and used to treat for five days cultures of striatal neurons derived from the same selection of lines. These cultures are a heterogenous population of mature striatal neurons, more immature GABA-ergic striatal neurons and proliferating progenitor cells. Various markers were therefore assessed to determine whether the proportion of any particular population was altered, suggesting a loss of cells of specific identity. However, no differences in the total numbers of cells, or the proportions of cells expressing βIII-tubulin (a marker of neuronal identity), CTIP2 (expressed by early GABAergic striatal neurons), DARPP32 (expressed by mature striatal medium spiny neurons) or nestin (expressed by neural stem cells), were observed, either on the basis of the *HTT* genotype of either cell type, or the stimulation of microglia with LPS (**Supplementary Fig. 9, Additional file** **2**)**.** Analysis of caspase 3 expression (a marker of apoptosis), or of nuclear γH2AX puncta (a marker of DNA damage and genotoxic stress; **Supplementary Fig. 10, Additional file** **2**), also did not reveal any microglial mutant HTT-dependent differences or an effect of their LPS stimulation (Fig. 6). Of note though, perhaps, numbers of γH2AX puncta were diminished in striatal neurons expressing HTT with expanded polyglutamine repeat lengths, irrespective of the presence or otherwise of conditioned supernatants. This is counter-intuitive, perhaps, but may suggest an inherent impairment of DNA repair mechanisms in these cells [48, 49]. Taken together, however, this suggests that the inflammatory molecules secreted at elevated levels by cultured HD microglia are insufficient to cause overt ill effects in PSC-derived striatal neurons, and/or that neurons in culture are in an environment sufficiently protective against insults of the magnitude generated by HD microglia.

## Discussion

Activated microglia and elevated plasma pro-inflammatory cytokine levels are observed in HD gene carriers, even in the pre-manifest stages of the disease [6, 21]. HD peripheral blood mononuclear cells are the likely source of the increased circulating levels of pro-inflammatory cytokines, as monocytes isolated from patients with HD and stimulated with lipopolysaccharide produce significantly more IL-6, IL-8 and TNFα compared with control subjects [27]. Here, we show that microglia, their tissue-resident CNS counterparts, are similarly hyper-responsive. Prior evidence obtained using transgenic mice has suggested the possibility of there being substantial overlap in the effects of disease-causing mutant HTT on peripheral monocytes and macrophages on the one hand, and microglia on the other [6, 15, 25]. The data presented here, however, shows for the first time that this is the case in microglia of human origin.

Crucially, this hyper-responsive phenotype must be due to the cell-autonomous effects of mutant HTT expression. That is, the cells are hyper-responsive not as a mere downstream effect of being in a disease environment, but because they themselves express mutant HTT. Previous observations both in primary monocyte-derived cells in which mutant HTT expression levels had been lowered and in a human myeloid cell line in which expression of mutant HTT fragments was induced indicated this to be the case for human myeloid cells in the periphery [27, 28]. Experiments in murine microglial models suggested the likelihood that this would be true for microglia too [16, 19, 25, 26]. The data shown here, in which disease-associated *HTT* CAG expansion mutations are expressed in cells that are naïve to the disease environment (this is true of the IsoHD ESC series, in particular), shows that this is indeed also the case for primary-like human microglial cells. Taken together, this is consistent with the possibility that exaggerated microglia activation might contribute to the inflammation observed early in the course of HD.

Given that the hyper-responsiveness observed in both human monocytes and microglia appears to be mutant HTT-dependent, it stands to reason that the processes underlying this phenotype are likely to be similar too. Large-scale transcriptomic studies of both human HD monocytes and murine mutant HTT fragment-expressing microglia-like cells have suggested that pro-inflammatory gene expression is elevated such that the cells are ‘primed’ to give an enhanced response to exogenous stimulation [26, 29]. The results described here provide a basis on which now to study further the mechanism(s) by which this occurs in human microglia.

Given the novel PSC-derived nature of these HD microglia, such future work depends on understanding whether expression of the mutant HTT affects per se the differentiation of the stem cells to their microglial fate. Using a panel of protein and gene markers, in which expression was compared with undifferentiated PSCs to confirm successful differentiation and to primary human monocytes to demonstrate their tissue-resident macrophage-like as opposed to monocyte-like characteristics, the present data showed no overt effect of the presence of an expanded HTT polyglutamine tract on the differentiation of these cells. This suggests that if the expression and activity of myeloid lineage-determining factors such as PU.1, C/EBPα and C/EBPβ are altered in differentiating human HD PSC-derived cells, as they are in mutant HTT fragment-expressing mouse microglial-like cells [26], the effects are not sufficient to overtly affect the overall maturity of the cells generated, at least in this PSC context. This is in contrast, perhaps, with reports of differences relating to neural development from mutant HTT-expressing human PSCs [50, 51]. Differential expression and binding of such factors, however, could remain a driver of the hyper-reactive, pro-inflammatory nature of human HD PSC-derived microglia.

Taken together, then, the data herein are consistent with a model in which mutant HTT promotes the expression of genes encoding pro-inflammatory mediators in microglia under basal conditions, a state in which the cells' responses to activating stimuli are then exaggerated. In disease conditions, on-going pathology could provide the stimulus for such enhanced responses to be realised, by means of so-called sterile inflammation. This is a process by which inflammation is triggered in the absence of microorganisms and is typically associated with the recognition by inflammatory signalling receptors of intracellular contents released from damaged and necrotic cells [52]. Such recognition occurs by both host pattern recognition receptors used in microbial detection, such as TLRs and NOD-like receptors, and other receptors that are not involved in pathogen detection. A resultant environment of persistent inflammation is unlikely one in which neurons will thrive, potentially adding to the stress that already vulnerable cells are undergoing in expressing the disease-causing mutation. That a parallel process of chronic, low-level inflammation also occurs in the periphery in HD might add to the effect as CNS and non-CNS components of the innate immune system communicate with each other as their responses progress. This systemic component may also, of course, contribute directly to known non-CNS pathology observed in the disease [53].

Elevated microglial activation may further compromise vulnerable neurons, or contribute to their loss, by means of the release of molecules that are toxic to them. Here, HD human PSC-derived microglia were shown to release elevated levels of reactive oxygen species, which directly cause stress and damage to neurons by way of their oxidative effects. This is in keeping with observations in animal models of transgenic HD mice, which show elevated reactive oxygen species production before the onset of symptoms, with lipid peroxidation appearing later [54, 55]. Oxidative stress may begin to cause significant damage to the HD brain as it ages, as endogenous anti-oxidant mechanisms become less able to counteract the elevated levels being produced [56]. Indeed, elevated levels of lipid peroxidation, DNA strand breaks, cytoplasmic lipofuscin and the accumulation of oxidative markers in DNA, each caused by oxidative stress, occur in the HD brain [57], whilst levels of the antioxidants such as SOD and ascorbate are reduced in various experimental models. Interventions upregulating these antioxidants appear to attenuate mHTT-induced toxicity of neurons [58, 59] and some have been tested for clinical efficacy, although results to date have been mixed [60].

It might be envisaged that potentially toxic effects of the HD microglial secretome on neurons could be modelled by means of measuring the effects of microglial supernatants on neurons derived from the same PSC lines. Striatal neurons, which are known to be selectively vulnerable in HD, can be derived from human PSCs. Having used a validated protocol for the generation of cultures containing such cells [44], they were exposed to supernatants from the HD human PSC-derived microglia. No overtly detrimental effects were observed, in contrast to the increased apoptosis seen when primary mouse neurons are exposed to mouse knock-in model mutant HTT primary microglia [26]. This may reflect one or more underlying differences in the model used, such as the more exaggerated effects that might be expected from microglia in which the disease-associated protein contains a particularly long polyglutamine tract, increasing its aggregate proneness and toxic effects. It may also be that the neurons require direct exposure to the microglial cells, as opposed to just their supernatants, for detrimental effects to be induced. Even then, any mutant HTT-dependent effects might have to be of sufficient magnitude to outweigh the possible intrinsic dampening effect on the inflammatory responses of differentiated microglia by the presence of PSC-derived neurons [61], or might be masked by sufficient levels of neurotrophic protection provided by the somewhat heterogeneous nature of PSC-derived neuronal cultures. Alternatively, a lack of elevated nitric oxide release by HD PSC-derived microglia may be key. Nitric oxide released by mouse microglia is neurotoxic by means of oxidative stress caused by the generation of peroxynitrite [62], but human microglia release nitric oxide at levels much lower than that of their mouse counterparts, perhaps reducing their potential for neurotoxicity in vitro [63]. Nevertheless, given careful design, further such experiments could have promise, as could examining the effects of HD microglia on both developmental and adult neurogenesis.

Accompanying their hyper-reactive phenotype, HD human PSC-derived microglia also showed modestly elevated levels of apoptosis, a process typical of myeloid cells when they are activated [64]. This effect became more pronounced when the cells are exposed to exogenous stress, suggesting that the resilience of HD microglia might be somewhat compromised by existing in their mutant HTT-induced hyper-reactive state. Microglial apoptosis was also enhanced by exposure to supernatants from human PSC-derived striatal neurons that expressed HTT with the longest mutant polyglutamine tract and were exposed to stress, indicating the possible influence that the environment of the Huntington’s brain may have on microglia and their responses.

Collectively, therefore, the present findings reveal a role for mutant HTT in disrupting the function of human microglia, providing a basis on which to better understand the contribution that their activation might have on HD pathogenesis. That homogeneous cultures of human HD PSC-derived microglia, which arguably are as close an in vitro model as is possible of the same human cells in vivo, can be robustly generated provides a strong basis on which to study further the underlying actions of mutant HTT. Moreover, given the potential contribution of microglia activation and inflammation to HD pathogenesis, they offer a robust platform on which to adapt their use for the screening of agents that might modulate their actions to beneficial effect in the disease. This is especially true given the use of series of lines that are isogenic or genetically matched, in which the potentially spurious effects of human genetic heterogeneity are removed such that any observed differences can be ascribed with confidence to the disease mutation itself. Further studies will be required to establish the full extent to which mutant HTT in human microglia promotes neuropathology in HD.

## Conclusion

In conclusion, we demonstrate the successful differentiation of microglial-like cells from human HD PSCs. These cells produce elevated levels of pro-inflammatory cytokines when stimulated and reactive oxygen species that may be neurotoxic. They also demonstrate some loss of viability and resilience to stress. These data are supportive of a model in which mutant HTT alters the basal state of microglia such that they are ‘primed’ to exaggerated responses to activating stimuli. The developing pathology of the HD brain could provide the stimulus for such enhanced responses to be realised. The resulting chronic neuroinflammation may in turn contribute to the toxic load in the brain as the disease progresses.

## Supplementary Information

**Additional file 1.** comprises Supplementary tables 1-4.

**Additional file 2.** comprises Supplementary figures S1-S10.

## Data Availability

The datasets supporting the conclusions of this article are included within the article and its additional files.

## Abbreviations

ANOVA: Analysis of variance
BCA: Bicinchoninic acid assay
BDNF: Brain-derived neurotrophic factor
BMP: Bone morphogenetic protein
BSA: Bovine serum albumin
cDNA: Complementary deoxyribonucleic acid
CNS: Central nervous system
CSF: Cerebrospinal fluid
EDTA: Ethylenediaminetetraacetic acid
ELISA: Enzyme-linked immunosorbent assay
ESC: Embryonic stem cell
GABA: Gamma-aminobutyric acid
GDNF: Glial cell-derived neurotrophic factor
GM-CSF: Granulocyte macrophage colony-stimulating factor
HD: Huntington’s disease
HEPES: 4-(2-hydroxyethyl)-1-piperazineethanesulfonic acid
HTT: Huntingtin
IL: Interleukin
(i)PSC: (Induced) pluripotent stem cell
LDEV: Lactose dehydrogenase elevating virus
LDH: Lactate dehydrogenase
LPS: Lipopolysaccharide
M-CSF: Macrophage colony-stimulating factor
MSN: Medium spiny neuron
PBS: Phosphate-buffered saline
qPCR: Quantitative polymerase chain reaction
RIPA: Radioimmunoprecipitation assay buffer
RNA: Ribonucleic acid
ROCK: Rho-associated protein kinase
ROS: Reactive oxygen species
SCF: Stem cell factor
SOD: Superoxide dismutase
TBHP: Tert-butyl hydroperoxide
TLR: Toll-like receptor
TNF: Tumour necrosis factor
VEGF: Vascular endothelial growth factor
