# Supplementary material for: Human Huntington’s disease pluripotent stem cell-derived microglia develop normally but are abnormally hyper-reactive and release elevated levels of reactive oxygen species
Source: J Neuroinflammation. 2021 Apr 19;18:94. doi: 10.1186/s12974-021-02147-6 (PMC8054367; doi:10.1186/s12974-021-02147-6)
Supplement: Supplementary file 1 — Additional file 1. comprises Supplementary tables 1-4. [file 12974_2021_2147_MOESM1_ESM.pdf]

Additional file 1

Supplementary Table 1

Pluripotent stem cells used in the study

PSC line	PSC series	Type	HTT polyQ-length	Origin
30^{Q}	IsoHD	ESC	30/19	Courtesy of M. Pouladi, Agency for Science, Technology and Research and National University of Singapore, Singapore
45^{Q}			45/19	
81^{Q}			81/19	
QS1	HD family	iPSC	58/22	UCL Queen Square Institute of Neurology
QS2			20/22	
QS3			69/22	
QS4			75/22	
QS5	-	iPSC	125/22	UCL Queen Square Institute of Neurology
15^{Q}	-	iPSC	15/15	Courtesy of H. Houlden, UCL Queen Square Institute of Neurology

Supplementary Table 2

Oligonucleotide primers used for qPCR

Gene target	Forward primer sequence	Reverse primer sequence
C1QA	GTGACACATGCTCTAAGAAG	GACTCTTAAGCACTGGATTG
GAS6	CGAAGAAACTCAAGAAGCAG	AGACCTTGATCTCCATTAGG
GPR34	GAAGACAATGAGAAGTCATACC	TGTTGCTGAGAAGTTTTGTG
PROS1	AAAGATGTGGATGAATGCTC	TCACATTCAAAATCTCCTGG
MERTK	AGGACTTCCTCACTTTACTAAG	TGAACCCAGAAAATGTTGAC
P2RY12	AAGAGCACTCAAGACTTTAC	GGGTTTGAATGTATCCAGTAAG
TMEM119	AGTCCTGTACGCCAAGGAAC	GCAGCAACAGAAGGATGAGG
TREM2	TCTGAGAGCTTCGAGGATGC	GGGGATTTCTCCTTCAAGA
CD93	CAGAATGCGGCAGACAGTTA	TTCAGCAGTCTGTCCCAGGT
CX3CR1	GGCAGACTTGGATTTCAGGA	GGGAACTGATCCATGGTGAA
CD14	ATTTGGTGGCAGGAGATCAA	GGCTTCCAGGCTTCACACT
HTT	AGTGATTGTTGCTATGGAGCGG	GCTGCTGGTTGGACAGAAACTC
GAPDH	AACAGCGACACCCACTCCT	CATACCAGGAAATGAGCTTGACAA
ACTB	AAGGCCAACCGTGAAAAGAT	GTGGTACGACCAGAGGCATAC

NANOG	AACTCTCCAACATCCTGAACCT	GGCATCCCTGCGTCACA
POU5F1	CTTCAGGAGATATGCAAAGCAGAA	CTGGGCGATGTGGCTGAT
SOX2	GAAATGGGAGGGGTGCAAAA	TACCGGGTTTTCTCCATGCT
ZFP42	CTCCCTTGGGAATTCAGACCTA	TCCAGCCTCAGTACATTTGTTTC

Supplementary Table 3

Antibodies used for immunofluorescence measures.

Antibody	Species	Dilution	Supplier
Anti-IF1/IBA1 (cat. NB100-1028)	Goat polyclonal	1:500	Novus Biologicals
Anti-PU.1 (cat. 2266)	Rabbit polyclonal	1:100	Cell Signaling Technology
Anti-TREM2 (cat. AF1828)	Goat polyclonal	1:50	R&D Systems
Anti-TMEM119 (cat. HPA052650)	Rabbit polyclonal	1:200	Atlas Antibodies
Anti-CTIP2 (25B6)	Rat monoclonal	1:300	Abcam
Anti-cleaved caspase 3 (5A1E)	Rabbit monoclonal	1:400	Cell Signaling Technology
Anti-DARPP32 (H-62)	Rabbit polyclonal	1:200	Santa Cruz Biotechnology
Anti-βIII-tubulin (cat. ab18207)	Rabbit polyclonal	1:500	Abcam
Anti-nestin (10C2)	Mouse monoclonal	1:600	Merck Millipore
Anti-gamma H2A.X (cat. ab11174)	Rabbit polyclonal	1:200	Abcam

Antibody	Supplier
Goat anti-rat AlexaFluor 488	Thermo Fisher Scientific
Goat anti-rabbit AlexaFluor 488	Thermo Fisher Scientific
Goat ant-mouse AlexaFluor 568	Thermo Fisher Scientific
Goat anti-chicken AlexaFluor 647	Thermo Fisher Scientific
Donkey anti-goat AlexaFluor 488	Thermo Fisher Scientific
Donkey anti rabbit AlexaFluor 568	Thermo Fisher Scientific

Supplementary Table 4

Perkin Elmer Columbus software parameters used for the unbiased analysis of each immunofluorescence imaging metric in the study.

Cell feature	Columbus building block	Specifics	Method	Output name
**Nuclei**	Find Nuclei	Channel: HOECHST 33342<br>Region: None	Method: M<br>Diameter: 15 µm<br>Splitting coefficient: 0.4<br>Common threshold: 0.1	Population: Nuclei
	Calculate Intensity Properties	Channel: HOECHST 33342<br>Population: Nuclei<br>Region: Nucleus	Method: Standard, mean	Properties: Intensity nuclear HOECHST 33342
	Calculate Morphology Properties	Population: Nuclei<br>Region: Nucleus	Method: Standard, area, roundness	Properties: Nucleus morphology
	Select Population	Population: Nuclei	Method: Filter by property<br>Nucleus area [µm²]: >= 40<br>Intensity nucleus HOECHST 33342 mean: <= 18512<br>Nucleus area [µm²]: <= 200<br>Boolean operations: F1 and F2 and F3	Population: Viable nuclei
**Caspase 3 +ve neurons**	Find Cytoplasm	Channel: Alexa 488<br>Nuclei: Nuclei	Method: B<br>Common threshold: 0.45<br>Individual threshold: 0.15	
	Calculate Intensity Properties	Channel: Alexa 488<br>Population: Nuclei<br>Region: Cytoplasm	Method: Standard, mean	Properties: Caspase 3 cytoplasmic intensity in all cells

	Select Population	Population: Nuclei	Method: Filter by property<br>Intensity cytoplasmic caspase 3 in all cells, mean: >= 1000	Population: Cells with cytoplasmic caspase 3 over a threshold
**Caspase 3 +ve microglia**	Find Cytoplasm	Channel: Alexa 488<br>Nuclei: Nuclei	Method: B<br>Common threshold: 0.45<br>Individual threshold: 0.15	
	Calculate Intensity Properties	Channel: Alexa 568<br>Population: Nuclei<br>Region: Cytoplasm	Method: Standard, mean	Properties: Caspase 3 cytoplasmic intensity in all cells
	Select Population	Population: Nuclei	Method: Filter by Property<br>Caspase 3 cytoplasmic intensity in all cells<br>Mean: >= 335	Population: Cells with Caspase 3 over a threshold
**Nestin+ cells**	Find Cells	Channel: Alexa 568<br>ROI: Viable nuclei<br>ROI Region : Cell	Method: B<br>Common threshold: 0.4<br>Area: > 70 μm^2<br>Split factor: 7<br>Individual threshold: 0.5<br>Contrast: > 0.3	Population: Nestin+ cells
	Find Cytoplasm	Channel: Alexa 568<br>Nuclei: Viable nuclei	Method: B<br>Common threshold: 0.45<br>Individual threshold: 0.15	
	Calculate Intensity Properties	Channel: Alexa 568<br>Population: Viable nuclei<br>Region: Cytoplasm	Method: Standard, mean	Properties: Intensity cytoplasm Alexa 568

	Select Population	Population : Viable nuclei	Method: Filter by Property<br>Intensity cytoplasm Alexa 568, mean: >= 1500	Population: Viable nuclei with cytoplasmic Nestin over a threshold
**βIII-tubulin+ neurons**	Find Cytoplasm	Channel: Alexa 647<br>Nuclei: Viable nuclei	Method: B<br>Common threshold: 0.45<br>Individual threshold: 0.15	
	Calculate Intensity Properties	Channel: Alexa 647<br>Population: Viable nuclei<br>Region: Cytoplasm	Method: Standard, mean	Properties: Intensity cytoplasm Alexa 647
	Select Population	Population: Viable nuclei	Method: Filter by Property<br>Intensity cytoplasm Alexa 647, mean: >= 1200	Population: Viable nuclei with cytoplasmic B3tubulin over a threshold
**CTIP2+ neurons**	Calculate Intensity Properties	Channel: Alexa 568<br>Population: Viable nuclei<br>Region: Nucleus	Method: Standard, mean	Properties : Intensity nucleus Alexa 568
	Select Population	Population: Viable nuclei	Method: Filter by property<br>Intensity nucleus Alexa 568 mean: >= 500	Population: CTIP+ cells
**DARPP32+ neurons**	Find Cells	Channel: Alexa 488<br>Region: None	Method: B<br>Common threshold: 0.4<br>Area: > 70 µm²<br>Split Factor: 7<br>Individual threshold: 0.5<br>Contrast: > 0.3	Population: DARPP32+ cells (Find Cells)
	Calculate Intensity Properties	Channel: Alexa 488<br>Population: DARPP32+ cells (Find Cells)	Method: Standard, mean	Properties: Intensity of DARPP32+ cells

		Region: Cell		
	Find Image Region	Channel: Alexa 488<br>Region: None	Method : Common threshold<br>Threshold: 0.5<br>Area: > 10 µm²	Population: DARPP32+ area<br>Region: DARPP32+ total staining
	Calculate Morphology Properties	Population: DARPP32+ area<br>Region: DARPP32+ total staining	Method: Standard, area, roundness	Properties: DARPP32+ total staining
	Calculate Intensity Properties	Channel: Alexa 488<br>Population: DARPP32+ area<br>Region: DARPP32+ total staining	Method: Standard, sum	Properties: Intensity DARPP32+ total staining
	Find Cytoplasm	Channel: Alexa 488<br>Nuclei: Viable nuclei	Method: B<br>Common threshold: 0.45<br>Individual threshold: 0.15	
	Calculate Intensity Properties	Channel: Alexa 488<br>Population: Viable nuclei<br>Region: Cell (2)	Method: Standard, mean	Properties: Intensity Cell Alexa 488
	Select Population	Population: Viable nuclei	Method: Filter by Property<br>Intensity Cell Alexa 488<br>Mean: >= 1200	Population: Viable nuclei with cytoplasmic DARPP32+ over a threshold
	Find Spots	Channel: Alexa 488<br>Region: Viable nuclei	Method: C<br>Radius: < 5px<br>Contrast: > 0.1<br>Uncorrected Spot to Region Intensity: > 1<br>Distance: > 3px<br>Spot Peak Radius: 0 px	Population: H2AX+ spots in viable nuclei

**H2AX+ spots**			Calculate Spot Properties	
	Select Population	Population: Viable nuclei	Method: Filter by property<br>Number of spots: > 4	Population: Viable nuclei with >4 H2AX+ spots
	Select Population	Population: Viable nuclei	Method: Filter by property<br>Number of Spots: > 10	Population: Viable nuclei with >10 H2AX+ spots
**TMEM119+ cells**	Calculate Intensity Properties	Channel: Alexa 568<br>Population: Viable nuclei<br>Region: Nucleus	Method: Standard, mean	Properties: Intensity Nucleus Alexa 568
	Select Population	Population:<br>Viable<br>nuclei	Method: Filter by property<br>Intensity Nucleus Alexa 568<br>Mean: >= 380	Population: Tmem119+ve viable cells (nuclear stain)
**IBA+ cells**	Find Cytoplasm	Channel:<br>Alexa 488<br>Nuclei: Viable Nuclei	Method: B<br>Common Threshold: 0.45<br>Individual Threshold: 0.15	
	Calculate Intensity Properties	Channel:<br>Alexa 488<br>Population: Viable Nuclei<br>Region: Cytoplasm	Method: Standard, mean	Properties: Intensity Cytoplasm Alexa 488
	Select Population	Population: Viable Nuclei	Method: Filter by Property<br>Intensity Cytoplasmic Alexa 488 Mean: >= 300	Population: Viable nuclei with cytoplasmic IBA1 over a threshold

**PU.1+ cells**	Calculate Intensity Properties	Channel: Alexa 568<br>Population: Viable Nuclei<br>Region: Nucleus	Method: Standard, mean	Properties: Intensity Nucleus Alexa 568
	Select Population	Population:<br>Viable Nuclei	Method: Filter by Property<br>Intensity Nucleus Alexa 568<br>Mean: >= 380	Population: PU1+ viable nuclei
**TREM2+ cells**	Find Cytoplasm	Channel: Alexa 488<br>Nuclei: Viable nuclei	Method: B<br>Common Threshold: 0.45<br>Individual Threshold: 0.15	
	Calculate Intensity Properties	Channel: Alexa 488,<br>Population: Viable Nuclei Region: Cytoplasm	Method: Standard, mean	Properties: Intensity Cytoplasm Alexa 488
	Select Population	Population: Viable Nuclei	Method: Filter by Property<br>Intensity Cytoplasm Alexa 488<br>Mean: >= 200	Population: Viable nuclei with cytoplasmic Trem2+ over a threshold
