# Supplementary material for: Human Huntington’s disease pluripotent stem cell-derived microglia develop normally but are abnormally hyper-reactive and release elevated levels of reactive oxygen species
Source: J Neuroinflammation. 2021 Apr 19;18:94. doi: 10.1186/s12974-021-02147-6 (PMC8054367; doi:10.1186/s12974-021-02147-6)
Supplement: Supplementary file 2 — Additional file 2. comprises Supplementary figures S1-S10. [file 12974_2021_2147_MOESM2_ESM.pdf]

Additional file 2

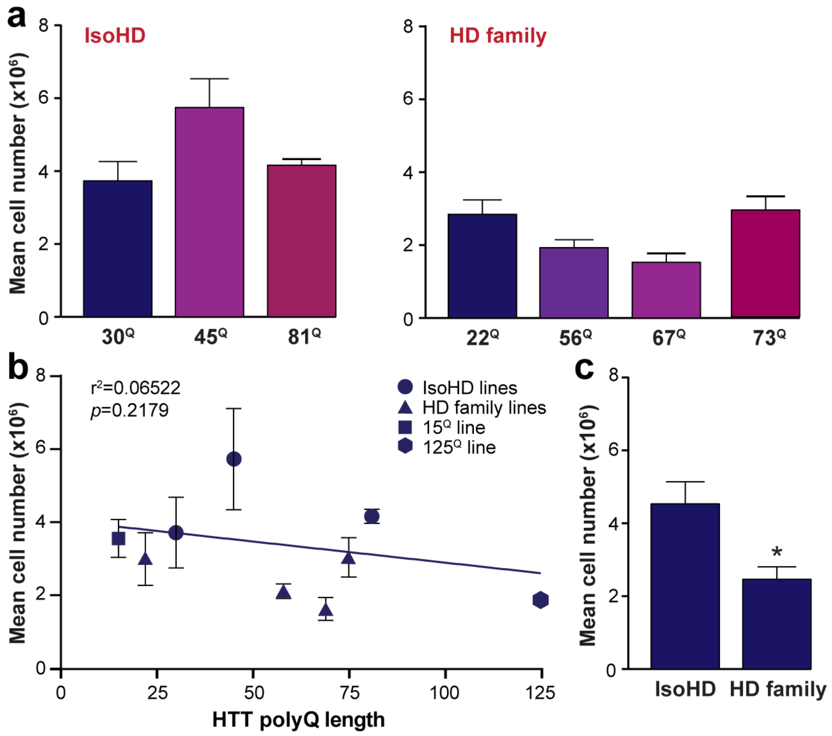

Supplementary Fig. 1. Mutant HTT does not affect the yield of cells obtained when differentiating human PSCs to a microglial fate

PSC-derived microglia of various HTT polyglutamine lengths were differentiated multiple times towards a microglial fate, as per published methods. For all lines, 150 embryoid bodies per differentiation were cultured in T175 flasks for three weeks to yield tissue-resident macrophage precursor cells. These were harvested at weekly intervals, usually between weeks four and six, and matured in culture for a further 14 days in the presence of 100 ng/ml GM-CSF and 100 ng/m IL-34. The total numbers of cells in the cultures from each harvest were counted and a mean of the yields per harvest determined for each differentiation. (**a**) Plotting these data for the lines belonging to the IsoHD ESC and HD family iPSC lines showed no significant differences in mean cell number between lines expressing wild-type (30^Q and 22^Q, respectively) and mutant (45^Q and 81^Q, and 56^Q, 67^Q and 73^Q, respectively) HTT. Data are presented as mean ± SEM, n=3, analysed by one-way ANOVA with Tukey's multi-comparison post-test. (**b**) When these data from the IsoHD and HD family series were plotted together and with those of other lines used in the study of varying HTT polyglutamine-lengths, no relationship between HTT polyglutamine-length and

mean cell number was observed. Data are presented as mean ± SEM, analysed by linear regression. (**c**) Although not HTT polyglutamine-length dependent, some considerable variability in mean cell number between lines was apparent. Indeed, when the mean of the mean cell number of all lines belonging to a series was compared, this showed a significant difference between the IsoHD (n=3) and HD family (n=4) PSC series. Data are presented as mean ± SEM, analysed by two-tailed unpaired Student's *t*-test (*$p<0.05$).

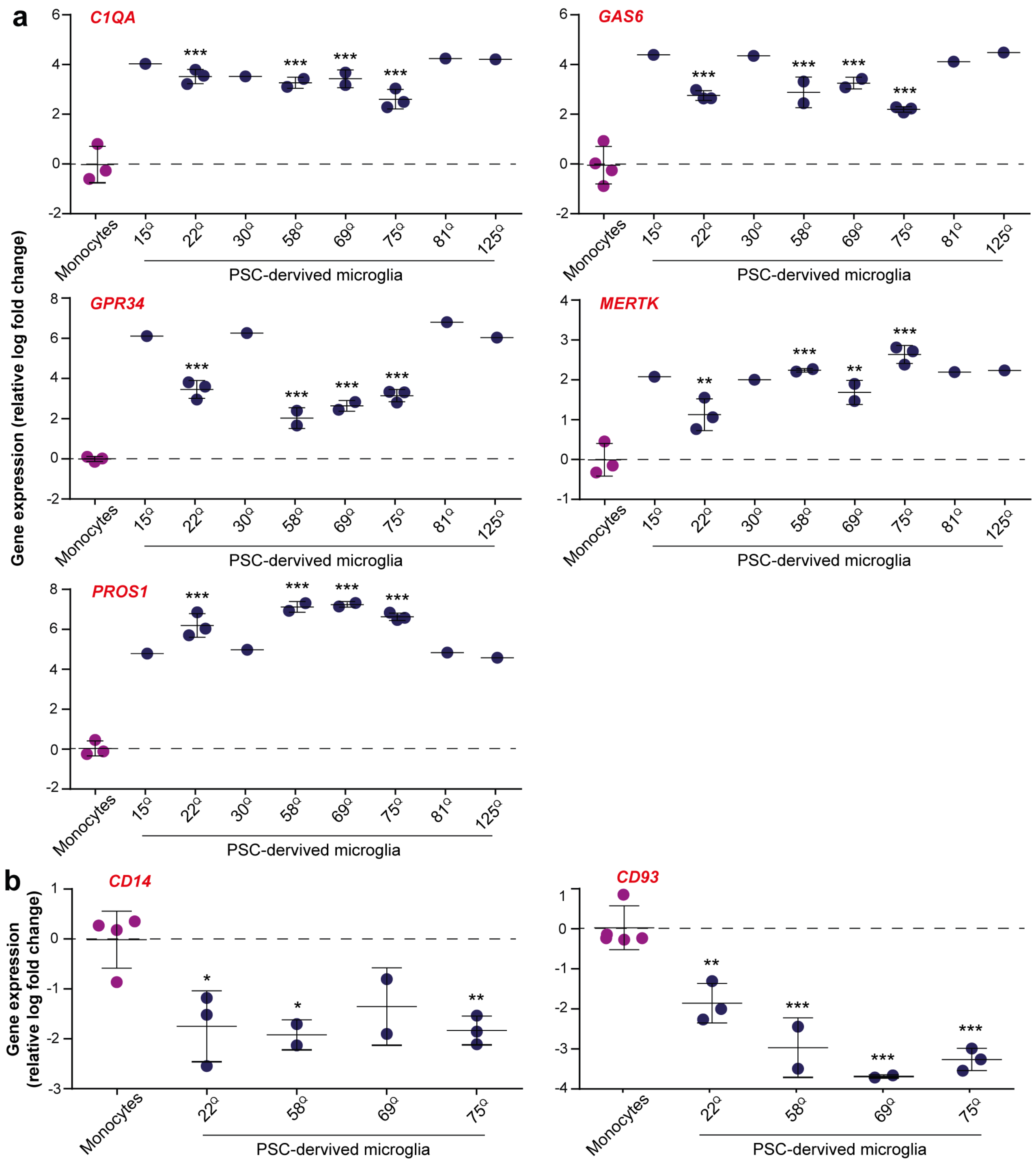

Supplementary Fig. 2. Mutant HTT does not affect the expression of a variety of genes associated with microglial identity in cultures derived from human PSCs

PSC lines expressing HTT of different polyglutamine repeat-lengths were differentiated to a microglial fate. (**a**) The identity of the cultures as tissue-resident macrophage/microglial-like of the myeloid lineage, as opposed to monocyte-like, was further demonstrated by qPCR assessment of the expression of other genes associated with microglial identity, *C1QA, GAS6, GPR34, MERTK* and *PROS1*, in the PSC-derived cultures as compared with primary human monocytes.

The aim was, again, both to confirm the robustness of the methods used across multiple lines and to test for any HTT polyglutamine repeat-length dependent effects on differentiation. Microglia of all lines showed the expected up-regulation of these genes compared with primary monocytes, but there were no mutant HTT repeat-dependent differences in their expression. (**b**) Similarly, two genes that are expected to be of lower expression in microglia compared with primary human monocytes, *CD14* and *CD93*, were assessed in a selection of the lines. These genes were indeed expressed at lower levels in the PSC-derived cells compared to primary monocytes. Data are presented as mean ± SEM, analysed by one-way ANOVA with Tukey's multi-comparison post-test, (* $p<0.05$, ** $p<0.01$, *** $p<0.001$). Some lines were tested only once; their data are included for illustrative purposes but were not included in the statistical analyses.

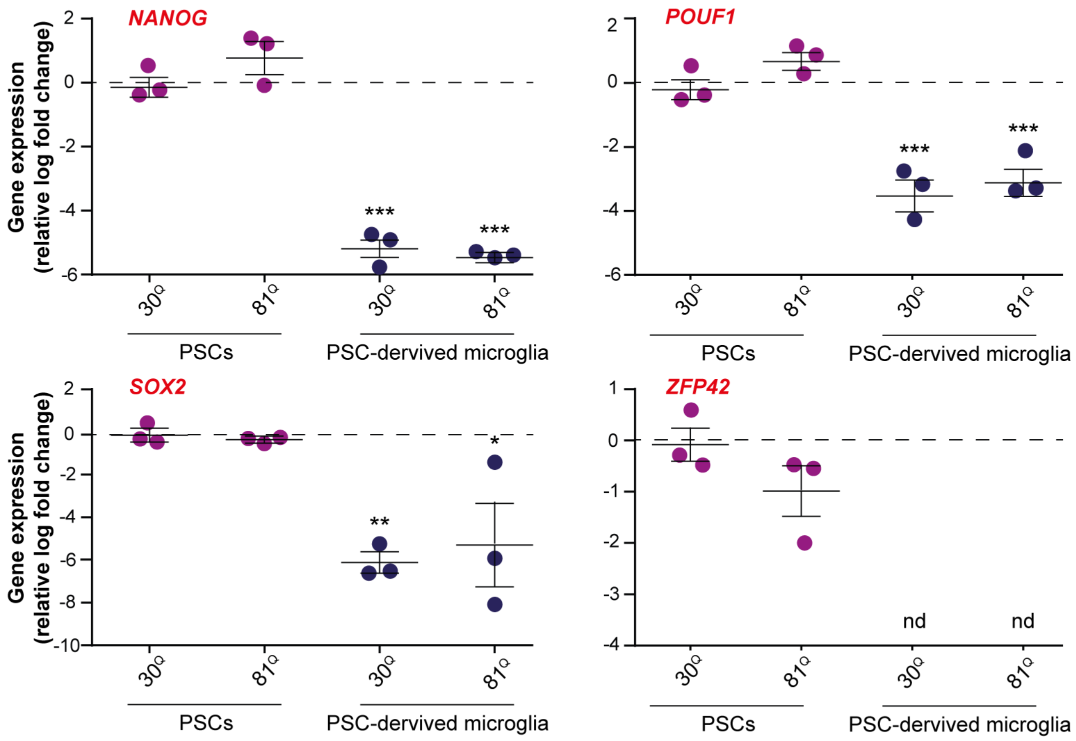

Supplementary Fig. 3. Pluripotency gene markers are downregulated in microglia derived from human PSCs irrespective of the presence of mutant HTT

IsoHD lines expressing HTT with either 30 or 81 polyglutamine repeats-lengths were differentiated to a microglial fate and their expression of the pluripotency marker genes, *NANOG*, *POUF1*, *SOX2* and *ZFP42*, was compared to the PSCs they were derived from. This showed the expected downregulation of all four genes in the microglial cultures, with no apparent mutant HTT-dependent differences. Data are presented as mean ± SEM, analysed by two-way ANOVA with Bonferroni post-test comparison of undifferentiated and differentiated cultures of the same IsoHD line (* p<0.05, ** p<0.01, *** p<0.001; n.d. = no detectable signal).

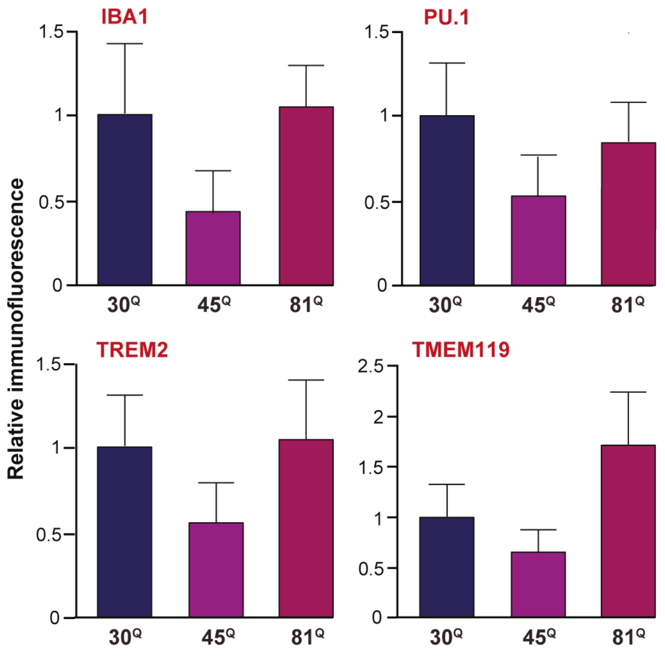

Supplementary Fig. 4. Mutant HTT does not affect the expression of key microglial marker proteins in human PSC-derived microglia

IsoHD PSC lines expressing HTT of different polyglutamine repeat-lengths were differentiated multiple times to a microglial fate. Quantification of the immunofluorescence signals of key protein markers of microglial identity was undertaken by high content immunofluorescence microscopy. This showed that the cultures, which were almost entirely enriched for microglial-like cells, showed no significant differences in the per cell intensity of the TREM2, IBA1, TMEM119 and PU.1 immunofluorescence signals that were measured. Data are presented as mean ± SEM, analysed by one-way ANOVA with Tukey's multi-comparison post-test.

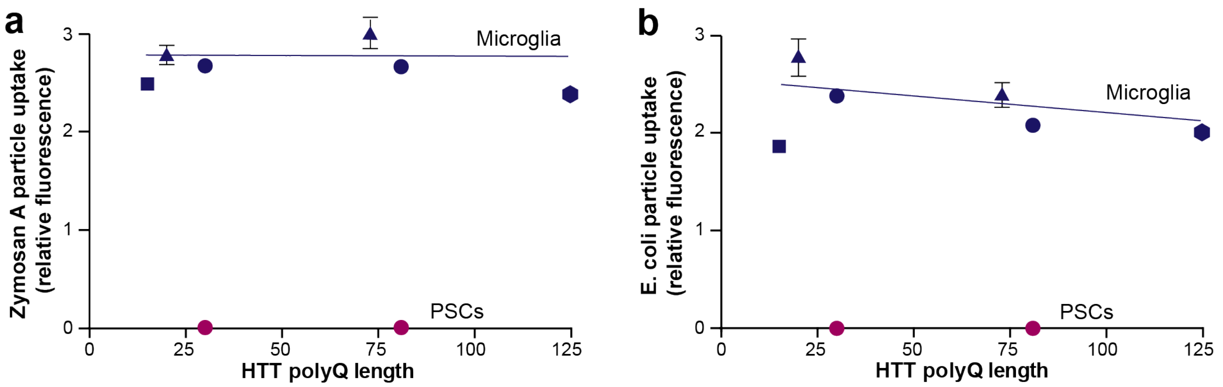

Supplementary Fig. 5. Phagocytosis does not appear to be altered in human Huntington's disease PSC-derived microglia

PSC-derived microglia of various HTT polyglutamine lengths were assessed for (**a**) zymosan A or (**b**) *E. coli*-mediated phagocytic uptake of pHrodo BioParticles. There were no HTT polyglutamine repeat-dependent differences in uptake by these cells. Data are presented as mean, ± SEM where multiple measurements of the same line were made, analysed by linear regression. The identity of the lines used are as follows: circles = lines of the IsoHD ESC series, triangles = lines of the HD family iPSC series, square = 1534 iPSC line, hexagon = QS5.1 line. IsoHD 30^Q and 81^Q lines were also analysed in their undifferentiated PSC state to demonstrate their absence of phagocytosis.

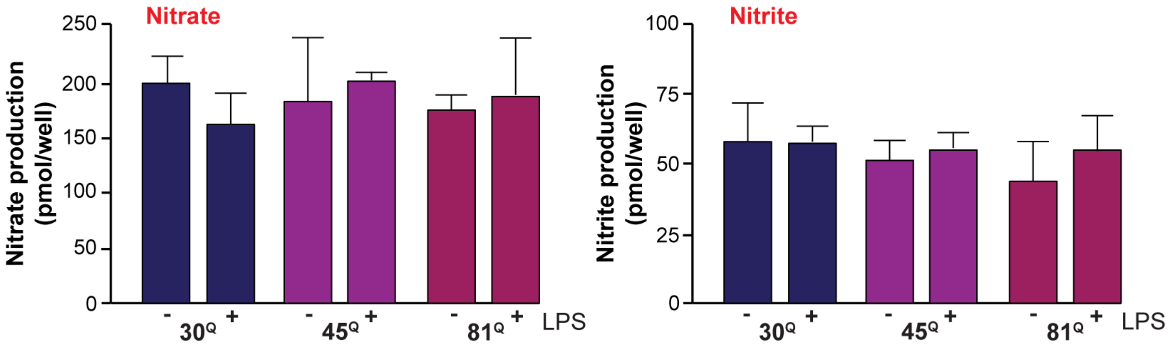

Supplementary Fig. 6. Human Huntington's disease PSC-derived microglia show no differences in the production of reactive nitrogen species

IsoHD PSC-derived microglia were assessed for production of reactive nitrogen species under unstimulated and stimulated (1 µg/ml LPS and 10 ng/ml IFNγ for 24 hr) conditions. There were no HTT polyglutamine repeat-dependent differences in nitrate or nitrite production by these cells. Data are presented as mean ± SEM, n=3, analysed by two-way ANOVA with Tukey's multiple comparisons test.

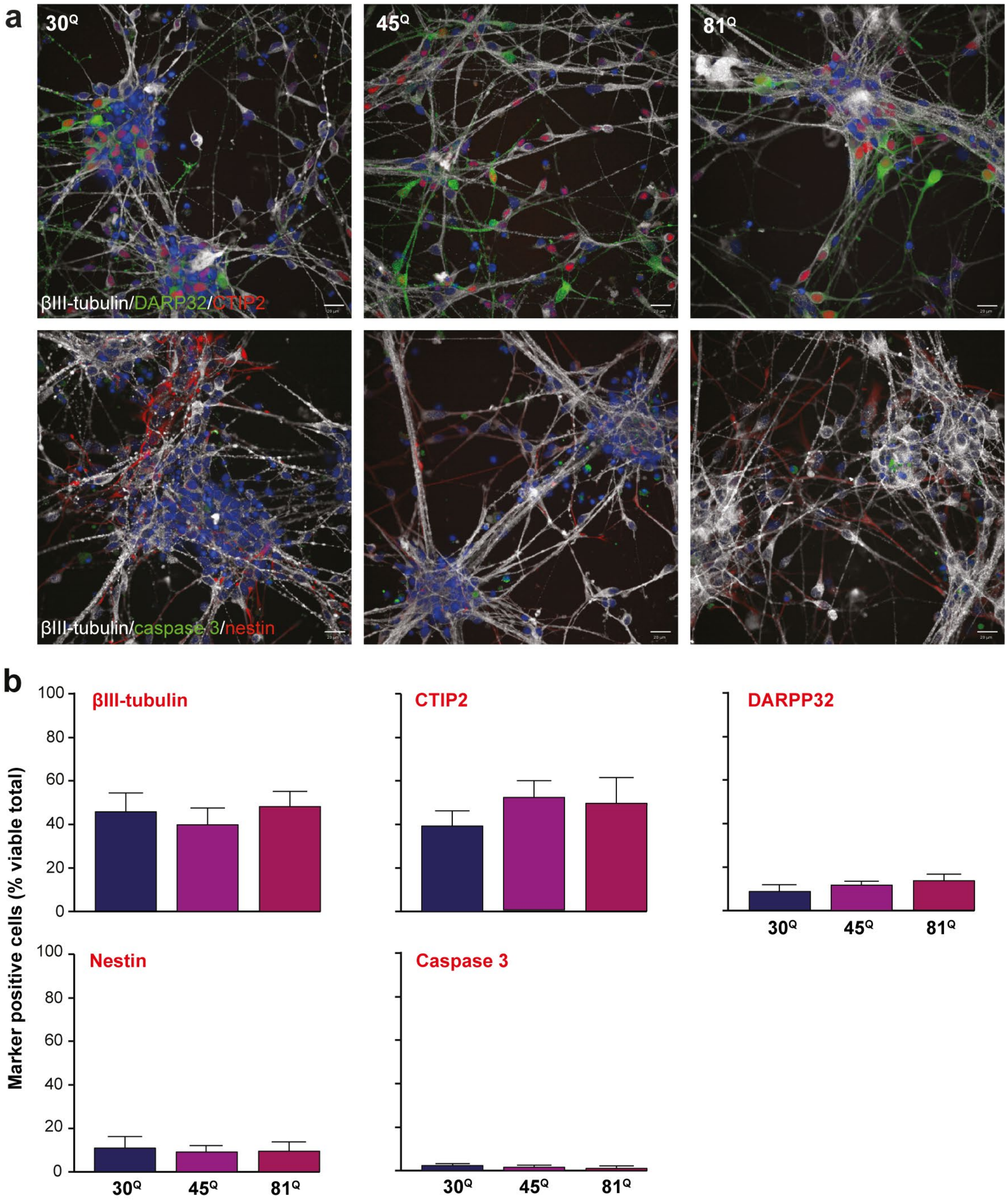

Supplementary Fig. 7. IsoHD ESCs produce DARPP32+ CTIP2+ striatal medium spiny neurons

(**a**) IsoHD ESCs expressing HTT with 30 ^{Q}, 45 ^{Q} or 81^{Q} generated cultures containing cells expressing the neuronal marker, βIII-tubulin, and the striatal medium spiny neuron markers, CTIP2 and DARPP32, as well as residual numbers of cells expressing the stem cell marker, nestin, as shown by confocal immunofluorescence microscopy. (**b**) High content imaging analysis showed no HTT polyglutamine-dependent effects on the proportions of cells expressing these

markers, or of a marker of apoptosis, caspase 3. Data are presented as mean ± SEM, n=3, analysed by one-way ANOVA with Tukey's post-test.

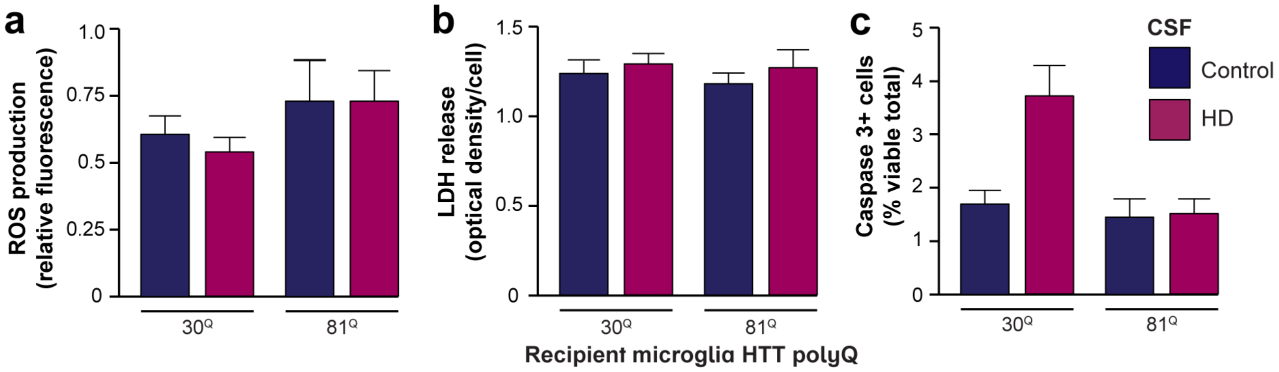

Supplementary Fig. 8. CSF from Huntington's disease patients does not have any overt effects on human PSC-derived microglia

IsoHD PSC-derived microglia were treated with CSF from HD and control human volunteers for 24 hr. HD CSF did not induce elevated (**a**) ROS production, (**b**) LDH release, or (**c**) caspase 3 activation in mutant HTT-expressing microglia. Data are presented as mean ± SEM, n=10 subjects for each group, analysed by two-way ANOVA with Tukey's multiple comparisons test.

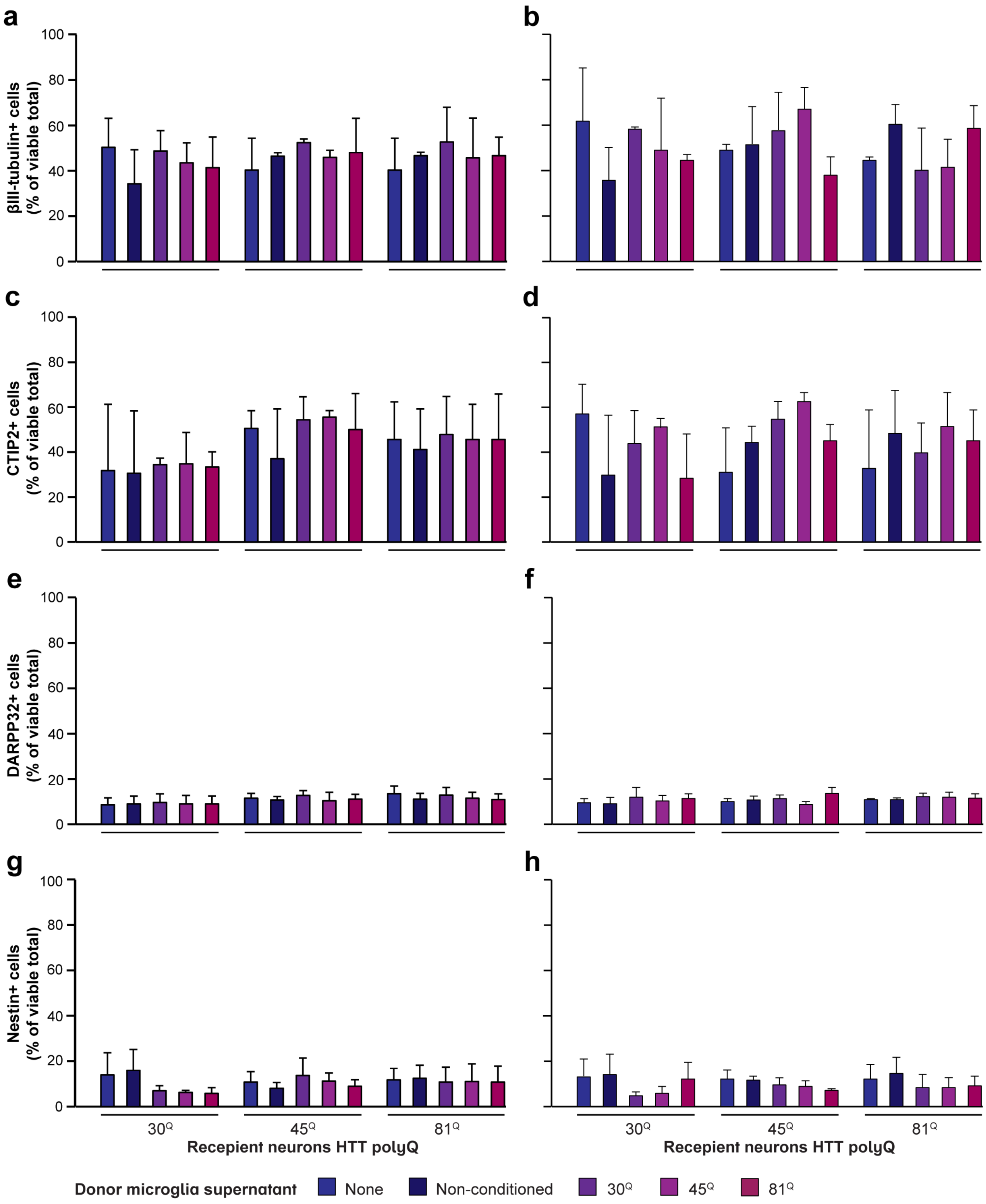

Supplementary Fig. 9. Human Huntington's disease PSC-derived microglia conditioned media do not affect the proportions of cells expressing various cell markers in cultures of PSC-derived striatal neurons

IsoHD PSC-derived striatal medium spiny neurons were cultured with supernatants harvested from IsoHD PSC-derived microglia. Numbers of recipient cells positive for (**a, b**) βIII-tubulin, (**c,**

d) CTIP2, (**e, f**) DARPP32, or (**g, h**) nestin, were not affected by exposure to conditioned media from microglia expressing mutant HTT for five days, either when (**a, c, e, g**) the microglia were unstimulated, or (**b, d, f, h**) had been activated by treatment with 1 μg/ml LPS and 10 ng/ml IFNγ for 24 hr. Data are presented as mean ± SEM, n=3, analysed by two-way ANOVA with Tukey's multiple comparisons test.

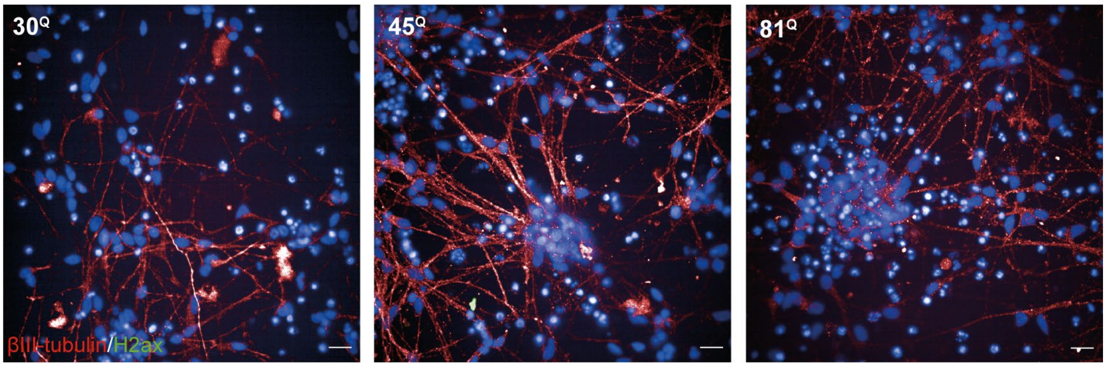

Supplementary Fig. 10. H2ax expression in IsoHD striatal medium spiny neurons

IsoHD PSC-derived striatal neurons expressing HTT of various polyQ repeat lengths show the presence of H2ax in their nuclei, as shown by confocal immunofluorescence microscopy.
